# Current status of sorafenib nanoparticle delivery systems in the treatment of hepatocellular carcinoma

**DOI:** 10.7150/thno.54822

**Published:** 2021-03-13

**Authors:** Fan-Hua Kong, Qi-Fa Ye, Xiong-Ying Miao, Xi Liu, Si-Qi Huang, Li Xiong, Yu Wen, Zi-Jian Zhang

**Affiliations:** 1 Department of General Surgery, The Second Xiangya Hospital, Central South University, Changsha, Hunan, China.; 2 Institute of Hepatobiliary Diseases of Wuhan University, Transplant Centre of Wuhan University, Zhongnan Hospital of Wuhan University, Wuhan University, Wuhan, Hubei, China.; 3 Department of Gastrointestinal Surgery, The Third Xiangya Hospital of Central South University, Changsha, Hunan, China.; 4 Department of Integrated Traditional Chinese & Western Medicine, The Second Xiangya Hospital, Central South University, Changsha, Hunan, China.

**Keywords:** Hepatocellular carcinoma, Sorafenib, Nanomaterials, Nanoparticles, Nanomedicine.

## Abstract

Hepatocellular carcinoma (HCC) is the most common type of liver cancer and one of the leading causes of cancer-related death worldwide. Advanced HCC displays strong resistance to chemotherapy, and traditional chemotherapy drugs do not achieve satisfactory therapeutic efficacy. Sorafenib is an oral kinase inhibitor that inhibits tumor cell proliferation and angiogenesis and induces cancer cell apoptosis. It also improves the survival rates of patients with advanced liver cancer. However, due to its poor solubility, fast metabolism, and low bioavailability, clinical applications of sorafenib have been substantially restricted. In recent years, various studies have been conducted on the use of nanoparticles to improve drug targeting and therapeutic efficacy in HCC. Moreover, nanoparticles have been extensively explored to improve the therapeutic efficacy of sorafenib, and a variety of nanoparticles, such as polymer, lipid, silica, and metal nanoparticles, have been developed for treating liver cancer. All these new technologies have improved the targeted treatment of HCC by sorafenib and promoted nanomedicines as treatments for HCC. This review provides an overview of hot topics in tumor nanoscience and the latest status of treatments for HCC. It further introduces the current research status of nanoparticle drug delivery systems for treatment of HCC with sorafenib.

## 1. Introduction

Liver cancer is the sixth most common cancer in the world 1, 2. The 5-year survival rate of patients with hepatocellular carcinoma (HCC) is only 18%, making it the second most fatal tumor after pancreatic cancer 3. HCC is the most common type of liver cancer and occurs mainly in China, Southeast Asia, and Sub-Saharan Africa 4. China accounts for the vast majority of HCC deaths in the world each year 5. Treatment options for HCC include hepatectomy, image-guided transcatheter tumor therapy, liver transplantation, transcatheter arterial chemoembolization (TACE), radiotherapy, chemotherapy, and combination therapy^6^. For early stage primary HCC (Barcelona Clinic Liver Cancer stage 0 or A), hepatectomy remains the first-line treatment. The mortality rate after surgery is low (<3%) 2, 7, 8. However, up to 70% of patients experience recurrence within 5 years 9. Currently, conventional antitumor drugs lack selectivity for tumor tissues 10-14, and the main obstacles to chemotherapy are multidrug resistance (MDR) and drug toxicity 15. Among solid tumors, HCC is considered a typical drug-resistant tumor, and strategies designed to overcome MDR are urgently needed 16-18. In recent years, progress in medical science and technology has facilitated development of numerous tumor molecule-targeted therapies, and drugs targeting specific molecules have shown good efficacy in treating HCC 19, 20. Molecule-targeted drugs are more specific to tumor tissue and more effective in treating cancer cells than traditional chemotherapy drugs 21, 22.

Sorafenib (SOR) is a dual aryl urea multikinase inhibitor and the first molecule-targeted drug approved for clinical treatment of HCC 23, 24. SOR exerts strong antitumor and antiangiogenic effects. It not only directly inhibits tumor cell proliferation by blocking the cell signaling pathway mediated by Raf/MEK/ERK but also indirectly inhibits tumor cell growth by blocking tumor angiogenesis by inhibiting vascular endothelial growth factor receptor (VEGFR) and platelet-derived growth factor receptor (PDGFR) 25. SOR satisfactorily improves the survival rate of patients 26, 27. Although SOR is currently widely used, it still has some unfavorable side effects 28. For instance, non-specific uptake of SOR by normal tissues may lead to a series of adverse reactions, such as skin rash, diarrhea, elevated blood pressure, and redness of the palms or soles of the feet 29-31. In addition, SOR is poorly water soluble and rapidly cleared and metabolized, which leads to low absorption efficiency in tumor tissues 32, 33. Moreover, some patients have congenital resistance to SOR or acquire resistance after treatment. Although SOR exerts antimetastasis and antiproliferation effects through multiple targets, such as EGFR, Raf, and PDGFR, not all HCC tumors overexpress these targets. Some tumors do not depend on the above pathways during tumorigenesis, and some pathways are selectively downregulated due to tumor heterogeneity during treatment. This indicates that acquired or primary SOR resistance is the main obstacle for survival of patients with liver cancer 24.

Researchers have developed many new SOR nanocarriers to overcome drug resistance in HCC. Nanoparticles (NPs) for drug delivery applications are typically 5-200 nm in size 34, 35. In recent years, the literature related to NPs in the treatment of HCC has increased significantly, achieving great progress in the application of NPs to treat HCC. NPs loaded with SOR (SOR-NPs) have a high release efficiency and bioavailability and actively target tumor tissues 36-38. SOR-NPs have a small diameter and large surface area, which increases the solubility of SOR. Furthermore, the characteristics of SOR-NPs can be controlled to facilitate delivery to the target tumor tissue 39, 40. In addition, the zeta potential and other characteristics of NPs can be engineered to improve cellular response. For example, a high absolute zeta potential, indicating a high surface charge density, increases cancer cell death and improves the treatment efficiency of SOR-NPs 41. Furthermore, by controlling drug release, NPs effectively reduce the therapeutic dose and frequency of administration. NPs reduce the cytotoxicity and degradation rate of chemotherapy drugs 40. Moreover, many drug-loaded NPs are delivered to tumor tissues *in vivo* using magnetic fields, and drug release can be triggered by acidic tumor environments. By bypassing the physical and physiological barriers that block conventional drugs, SOR-NPs effectively treat cancer. Therefore, nanotechnology has the potential to alter the resistance of cancer cells to cancer drugs and overcome MDR 17.

**Figure 1:**
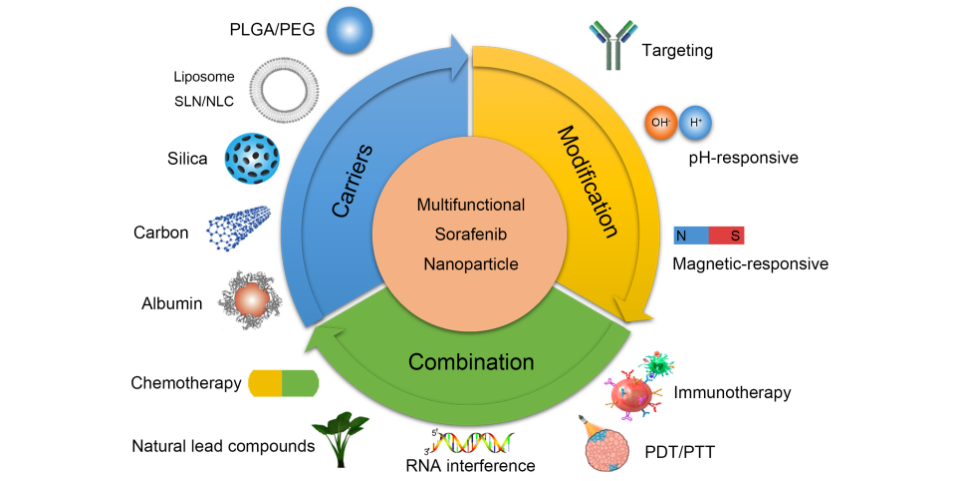
Research directions in the development of SOR-NPs for HCC. (Top left) Improving the biocompatibility of SOR using various nanocarriers. (Top right) Modification methods to increase the targeting and responsiveness of SOR-NPs. (Bottom) Enhancing treatment efficacy through combination therapies. HCC: hepatocellular carcinoma; PDT: photodynamic therapy; PEG: Polyethylene glycol; PLGA: poly (lactic-co-glycolic acid); PTT: photothermal therapy; NLC: nanostructured lipid carriers; NP: nanoparticle; SLN: solid lipid nanoparticles; SOR: sorafenib.

This review considers the limitations of SOR in treating HCC as a starting point and explains the current status of SOR-NP from the perspectives of biocompatibility, material properties, and combination therapy. This review focuses on polymer NPs, lipid NPs (LNPs), silicon NPs, and metal NPs and provides a brief introduction to multifunctional nanoplatforms loaded with SOR. By introducing the current status of SOR-NPS, we aim to inspire future research on liver cancer treatment. The graphical abstract is shown in Figure 1.

## 2. Enhancing the biocompatibility of SOR with NPs

NPs are attractive for medical applications due to their excellent stability, superior structural design, variable and controlled solubility, low immunogenicity, good cellular biocompatibility, antigenicity, three-dimensional geometric structure, and specific tissue/cell targeting abilities 42, 43. The modes of action of SOR-NPs include controlled release of amphiphilic drugs, continuous release of therapeutic agents, cyclic dosing, and passive delivery of drugs to tumor tissues through the enhanced permeability and retention (EPR) effect 44. In addition, active targeting of NPs, particularly via attachment of cancer tissue ligands to the NP surface, confirms the clinical efficacy of improved drugs 44, 45.

Metabolism kinetics are the main factors affecting the toxicity of NPs *in vivo*. Some physical and chemical properties of NPs (e.g., size, charge, and surface properties) directly affect their metabolism 46. The advent of nanomedicine has made poorly soluble drugs useful. Poorly soluble drugs can be entrapped, encapsulated, coated, or chemically bound in NPs 47. NPs have also been developed with favorable pharmacokinetics to avoid toxicity and side effects 48, to target the desired site of action, and to provide triggered drug release. The therapeutic outcome depends on the NP biodistribution, which is impacted by the tissue targeting approach. Passive targeting utilizes the disease physiology 49, whereas active targeting utilizes tailored surface coatings or conjugated ligands 50. Blood and lymphatic circulation both play crucial roles in the transport of NPs. Most uncoated NPs are cleared from circulation by the mononuclear phagocyte system. Pharmacokinetic and pharmacodynamic (PB/PKPD) indexes of SOR-NPs are summarized in Table 1.

### 2.1. Polylactic acid (PLA) and poly(lactic-co-glycolic acid) (PLGA) NPs

**Figure 2:**
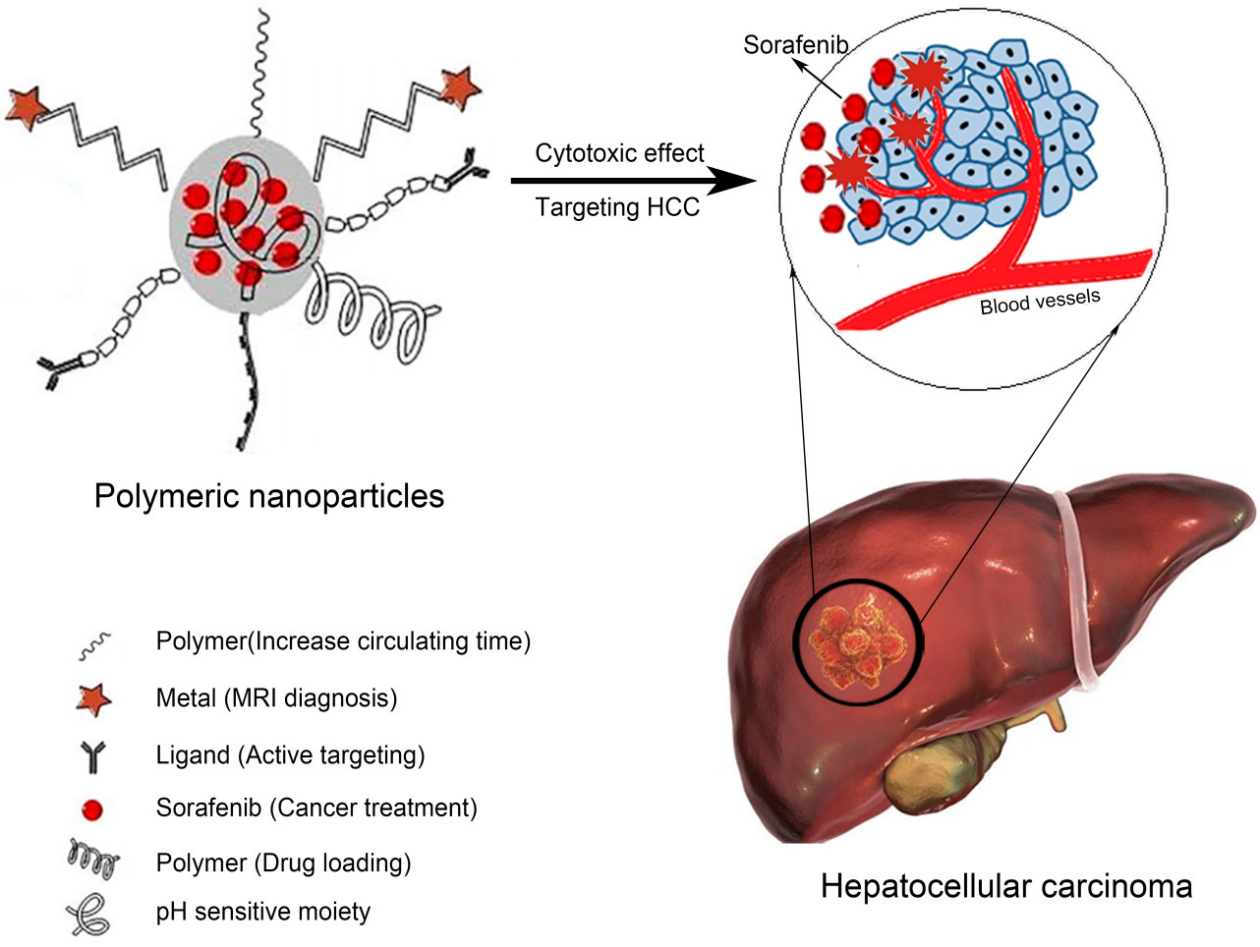
Structures of polymer SOR-NPs for targeted treatment of HCC. MRI: magnetic resonance imaging.

PLA is widely used to prepare polymer NPs due to its biodegradability, self-assembly properties, ability to actively target tumor tissues when attached to aptamers or antibodies, and utility in magnetic resonance imaging (MRI). For example, Craparo et al. synthesized polymer NPs through the sequential chemical reaction of α,β-poly(N-2-hydroxyethyl) (2-aminoethylcarbamate)-N/L-aspartamide (PHEA-EDA) with PLA and lactose^51^. After modification with anti-sialic acid glycoprotein receptor (ASGP-R), SOR was loaded by evaporation. The size of the resulting NPs was on the nanometer scale, and the zeta potential was slightly positive. Biodistribution studies in mice revealed significant accumulation of the NPs in the liver after oral administration. PLGA is a Food and Drug Administration (FDA)-approved biodegradable material that is widely used in cancer nanotechnology. It is suitable for encapsulating lipophilic drugs due to its hydrophobicity. In addition, the range of applications of PLGA has been expanded by optimizing its structure and increasing its drug loading rate. The characteristics of example polymer NPs are listed in Table 2. The structures of polymer SOR-NPs for targeted treatment of HCC are shown in Figure 2. Polymer NPs can limit drug intake to once a day, thus improving patient compliance. Therefore, polymer NP controlled-release systems are now being incorporated into the treatment of HCC 52.

### 2.2. Polyethylene glycol (PEG) NPs

Thermosensitive hydrogels, such as PEG, polycaprolactone (PCL), PLA, and polypropylene oxide (PPO) block copolymers, are ideal materials for continuous drug release. At temperatures below the lower critical solution temperature, these materials are in a sol state with a low viscosity, but they enter a gel state at higher temperatures 53. Thermosensitive hydrogels are promising drug carriers because of their biodegradability, low toxicity, high drug loading capacity, site specificity, sustained release, controlled release, and other advantages 54. For example, Zheng et al. synthesized a thermosensitive composite NP that was used as an effective drug carrier for SOR and was combined with radiotherapy for local and continuous treatment of liver cancer 55. The NPs continuously released SOR and displayed a prolonged hydrogel degradation time (more than 15 days), exerting site-specific and long-term anticancer effects. In addition, PEG reduces uptake of circulating NPs by the reticuloendothelial system and inhibits non-specific adsorption of proteins. These properties allow the NPs to circulate in the blood for longer periods, increasing the probability of accumulation in target tumor tissues 56. Wang et al. developed poly(L-glutamic acid)-graft-methoxy PEG/combretastatin A4 sodium salt NPs (CA4-NPs) encapsulating SOR as a collaborative treatment for HCC. CA4-NPs destroyed the established tumor blood vessels and induced extensive tumor necrosis, and SOR reduced VEGF-A-induced angiogenesis to further inhibit tumor proliferation and exert a synergistic effect with CA4-NPs. In subcutaneous and *in situ* liver tumor models, the group treated with the combination of CA4-NPs and SOR exhibited a significantly reduced tumor volume and prolonged survival compared with those treated with CA4-NPs or SOR alone 57.

Sheng et al. used a nanoprecipitation method to combine SOR with a PEG monomethyl ether-racemic PLA copolymer to synthesize SOR-NPs 58. Compared with free SOR, the retention time of the SOR-NPs increased significantly *in vivo*. A significantly higher concentration of SOR was observed in tumor tissues than in normal tissues. SOR-NPs were more effective in inhibiting tumor growth than SOR alone.

Monajatic et al. synthesized cholesterol-branched polyethyleneimine (PEI) lipid polymer NPs, as confirmed by infrared spectroscopy, NMR, and other methods 59. PEGylation reduced the size of the nanometer assembly from a rod to a sphere, and this change in particle morphology promoted cellular uptake of SOR and increased its cytotoxicity. Therefore, this NP both increased the cellular uptake and reduced the general cytotoxicity of SOR; thus, it is a potentially useful carrier for delivering poorly soluble chemotherapy drugs such as SOR to HCC tumors.

### 2.3. Liposomes

LNPs are one of the most popular bioactive nanocarriers for tumor treatment. LNPs have facilitated advances in the use of NPs to treat HCC by improving the therapeutic efficiency of antitumor drugs. LNPs include liposomes, solid lipid nanoparticles (SLNs), and nanostructured lipid carriers (NLCs) 60. LNPs have been loaded with lipophilic and hydrophilic drugs to extend their half-life and release time. Because the time of action of the drug in the tumor is prolonged, the therapeutic effect is enhanced 61. LNPs also display good stability, high drug loading rates, and are easy to prepare; thus, they can be produced at a large scale. In addition, encapsulation of chemotherapy drugs in LNPs not only reduces the side effects of the drugs but also reduces treatment resistance by increasing the drug concentration in tumor tissues and decreasing the concentration in normal tissues 60.

Liposomes are spherical vesicles containing water formed from a single or multiple lipid bilayers composed of amphiphilic phospholipids and cholesterol. Hydrophilic drugs are encapsulated in the inner aqueous phase, whereas lipophilic drugs are encapsulated in the lipid bilayer 62. The composition of liposomes is similar to physiological membranes. In addition, liposomes are non-toxic and biocompatible and exert significant effects on cell endocytosis 63. Liposomes have been widely used as drug carriers to improve the efficacy of chemotherapy drugs 64.

For example, Yang et al. developed lipid nanosuspensions loaded with SOR using a nanoprecipitation method to improve the efficacy of SOR in treating HCC 65. The NPs were spherical with a uniform size distribution. The *in vitro* cytotoxicity of the NPs towards HepG2 and Bel-7402 cells was greater than that of SOR. In an H22 tumor-bearing mouse model, the NPs significantly reduced tumor volume compared with SOR. Additionally, Zhang et al. developed hyaluronic acid (HA)/lipid hybrid SOR-NPs (HA/SOR-cLNs) 66. The NPs were spherical with a uniform particle size distribution and good blood compatibility and histological safety. The NPs were degraded in the presence of hyaluronidase, thus causing enzymatic release of SOR. Compared with SOR solution, the NPs were more cytotoxic. *In vivo* imaging experiments showed effective accumulation of HA-cLNs in tumor sites compared with cLNs and free DiR. *In vivo* antitumor effects indicated the superiority of HA/SOR-cLNs to all other treatments.

### 2.4. Solid lipid nanoparticles (SLNs)

SLNs are solid colloidal particles that carry natural or synthetic solid lipids, such as lecithin and triacylglycerol, with drugs wrapped or embedded in the lipid core. SLNs are characterized by site-specific targeting, good stability, and the ability to encapsulate lipophilic and hydrophilic drugs. Moreover, they are inexpensive, can be easily prepared, and have low toxicity. For example, Grillone et al. coated SOR and superparamagnetic iron oxide NPs (SPIONs) in a hexadecyl palmitate lipid matrix by thermal homogenization to obtain NPs that increased SOR delivery to tumor sites with the help of a distal magnetic field and reduced the effects of SOR on healthy tissues 67. The efficiency of SOR loading in the NPs was ~90%, SOR loading was very stable in water, and the NPs showed good cellular compatibility. The NPs substantially inhibited the proliferation of HepG2 tumor cells. Benizric et al. developed a new class of SLNs based on nucleoside lipids with positive or negative zeta potentials (SLNs+/-) depending on the charge of the lipid 68. The nanoparticles were composed of monocrystalline silicon and had a parallelepiped shape. The stability of the NPs was regulated by the lipids. Importantly, SLN+ and SLN- NPs significantly improved the water solubility of SOR (>120 μM), resulting in better antitumor effects. Tunki et al. prepared ligand-coupled SOR-SLNs (GAL-SOR-SLNs) 69. The SLNs were prepared by emulsion and solvent evaporation methods, labelled with galactose by combining the amine of PEGylated galactose with carboxyl groups on the SLNs to form an amide bond, and finally loaded with SOR. The GAL-SOR-SLNs exerted stronger cytotoxic effects and demonstrated better targeting and pharmacokinetic properties than SOR or SOR-SLNs.

### 2.5. Nanostructured lipid carriers (NLCs)

NLCs are second-generation LNPs consisting of solid and liquid lipids that were developed to overcome the limitations of SLNs. NLCs exhibit higher drug loading capacities than SLNs, and lipid crystallization is avoided during drug storage because of the presence of liquid lipids, thus preventing drug expulsion. The main advantages of NLCs are that they can be loaded with hydrophilic and hydrophobic drugs, surface modified, and targeted to specific sites, they controllably release drugs, and they show low toxicity *in vivo*. However, some deficiencies still exist. For example, the lipids are readily excreted after polymorphic transformation in the nanocarrier matrix, and the drug loading efficiency is still low and insufficient 70. Bondi et al. prepared NLCs using a mixture of solid lipids (tripalmitin) and liquid lipids (Captex 355 EP/NF or Miglyol 812; NLC-A/B) 71. This preparation achieved higher drug loading and longer storage stability than solid lipids. The obtained NPs were nanosized and had a high negative zeta potential. SOR-loaded NLC-A/B exhibited stronger antitumor activity than free SOR. Wang et al. fabricated NLCs to co-deliver doxorubicin (DOX) and SOR 72. With the combination of DOX-induced immunogenic cell death and SOR-mediated tumor microenvironment (TME) remodeling, the NPs showed strong anticancer benefits and immune response after treatment.

### 2.6. Silica NPs

**Figure 3:**
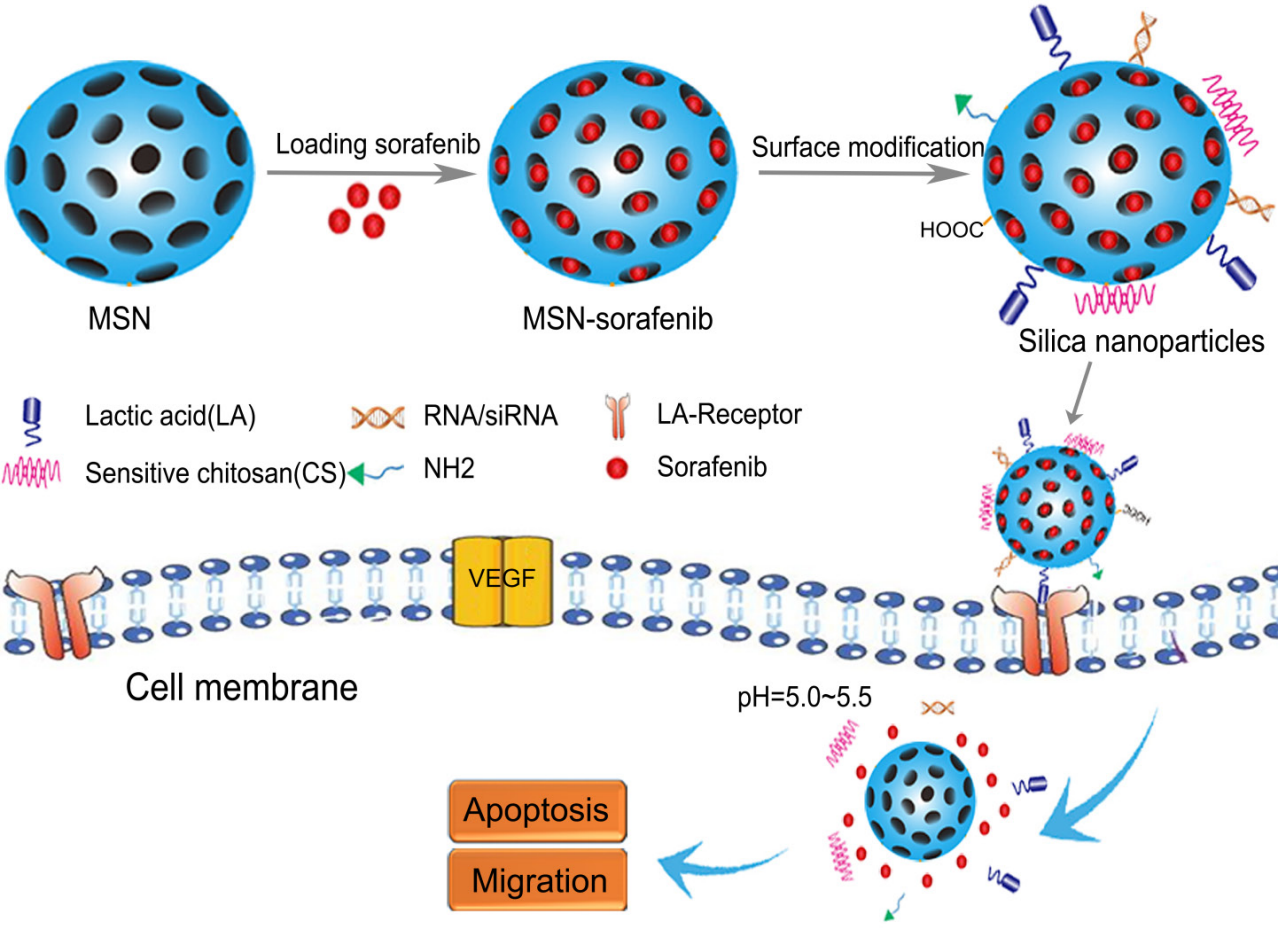
Structures of silica SOR-NPs modified with surface groups for targeted delivery and pH-responsive drug release in HCC. CS: sensitive chitosan; LA: lactic acid; MSN: mesoporous silica nanoparticles; NH2: amino; VEGF: vascular endothelial growth factor.

Silica NPs, particularly amorphous silica NPs, are widely used in medical applications for the delivery of chemotherapy drugs and multimodal imaging 73. Silica NPs have a hydrophilic multifunctional silane surface, which is conducive to long-term circulation, good biocompatibility, easy synthesis, and low production costs 74. In recent years, mesoporous silica NPs have attracted attention due to their large specific surface area, high porosity, tunable mesoporous structure, and easy surface modification. In particular, the drug loading efficiency of silica NPs is high because drugs can be encapsulated inside the pore channels. On the other hand, non-porous silica NPs are also used for drug delivery and play an important role in targeted molecular imaging, which is helpful for early diagnosis of diseases 75, 76. The characteristics of example silica NPs are listed in Table 3. As shown in Figure 3, silica SOR-NPs have been modified with surface groups for HCC targeting and pH-responsive release of SOR in tumor tissues.

### 2.7. Carbon nanomaterials

Carbon nanotubes (CNTs) are cylindrical tubes composed of graphene sheets with an overall needle-like shape that is open at one end and sealed at the other 77. Depending on the number of layers forming CNTs, these structures are divided into single-walled CNTs and multi-walled CNTs. Graphene is quite safe for cells, can promote neuron growth, and has long-term biocompatibility in the liver 77. Thus, in recent years, SOR-CNTs have been synthesized. For example, Elsayed et al. loaded SOR onto functionalized CNTs through physical adsorption and then microencapsulated the CNTs in alginate 78. *In vitro* proliferation studies revealed that the NPs had at least two-fold higher cytotoxicity toward HepG2 cells than SOR alone. In addition, the circulating α-fetoprotein heterogeneity ratio in a chemically induced model of HCC was significantly reduced by the NPs (14.0%) compared with no treatment (40.3%) or SOR (38.8%).

Xu et al. reduced graphene oxide (GO) in the presence of SOR using ascorbic acid as a green reducing agent to generate SOR-reduced GOs for the treatment of gastric cancer 79. The NPs had a transparent and smooth morphology and presented significant cytotoxicity against SGC7901 cancer cells. Thapa et al. prepared folic acid (FA)-conjugated polyvinyl pyrrolidone-functionalized GOs for the targeted delivery of SOR. The authors found that the NPs had improved SOR drug loading capacity and stability. The NPs enabled targeted delivery of SOR to cancer cells expressing high levels of FA receptors, thereby enhancing SOR release under acidic conditions. Although not all of the above-mentioned studies focused on liver cancer, it is conceivable that these materials may be effective against HCC 80.

### 2.8. Albumin

Albumin (66.5 kDa) is the most abundant plasma protein in the human body (35-50 g/L human serum). The versatility of albumin and its natural origin make it an ideal candidate for drug delivery applications. Albumin plays an increasingly important role as a clinical drug carrier. Three main drug delivery technologies have been reported: conjugation of low molecular weight drugs with exogenous or endogenous albumin, conjugation with bioactive proteins, and encapsulation of drugs in albumin NPs 81. For example, Wang et al. encapsulated SOR in bovine serum albumin (BSA) NPs and chemically modified them with FA 82. After optimization, the average particle size, zeta potential, drug encapsulation efficiency, and drug loading efficiency of the NPs were 158.00 nm, -16.27 mV, 77.25%, and 7.73%, respectively, and the formulation remained stable at room temperature for more than 1 month. In addition, Pascalau et al. prepared spherical core-shell microcapsules based on a BSA gel for targeted delivery of SOR to liver cancer 83. The microcapsules consisted of a composite multi-layer shell composed of polysaccharides with opposite charges (HA and chitosan) encapsulating SOR.

## 3. Targeted and responsive delivery of SOR with NPs

In recent years, researchers have built not only nanoplatforms to improve the biocompatibility of SOR but also nanoplatforms with active targeting capabilities, pH response, and magnetic field response, which has expanded their application prospects. These nanoplatforms have improved the solubility, tumor tissue retention, and therapeutic efficacy of SOR *in vivo*. These results have mainly been achieved by the following methods: (1) increasing the concentration of SOR entering tumor cells 84; (2) controlling the release of SOR in time/space 85; (3) real-time monitoring of SOR 86.

### 3.1. Targeted delivery of SOR

Targeted delivery of SOR-NPs is an effective method to improve treatment efficacy and reduce the toxicity of SOR to non-tumor cells. Tumor-targeting mechanisms are divided into two categories: passive targeting and active targeting. Passive targeting requires the EPR effect, which causes leakage of NPs into tumor tissue through abnormal and highly heterogeneous microvasculature and retention due to dysfunctional lymphatic drainage. By contrast, active targeting involves interactions between specific receptors overexpressed on target cells in tumor tissues and targeting modifiers bound on the surface of SOR-NPs 87. It is generally believed that the targeting effect of active strategies is more significant than that of passive targeting, but it is also more difficult to modify.

#### 3.1.1. Passive targeting

NP size plays a key role in passive targeting. Through the EPR effect, large NPs have good retention in blood vessels but are quickly cleared from tumor tissue after extravasating; by contrast, small NPs can penetrate deep into tumor tissues 49.

Cervello et al. designed polymer SOR-NPs from a brush copolymer synthesized by atom transfer radical polymerization of PHEA and poly(butyl methacrylate) 88. Due to the EPR effect, the NPs accumulated at significant levels in xenograft tumors through passive targeting and exerted enhanced anticancer effects. Khan et al. used poly(butadiene)-block-poly(ethylene oxide) to generate polymer SOR-NPs 89. According to DLS and cryo-TEM measurements, the NPs had a particle size of ~282 nm, PDI < 0.29, film thickness of ~20 nm, SOR encapsulation rate of 71%, and sustained SOR release for up to 144 h. The cytotoxicity of the SOR-NPs to HepG2 cells was 1.7 times higher than that of a SOR suspension.

**Figure 4:**
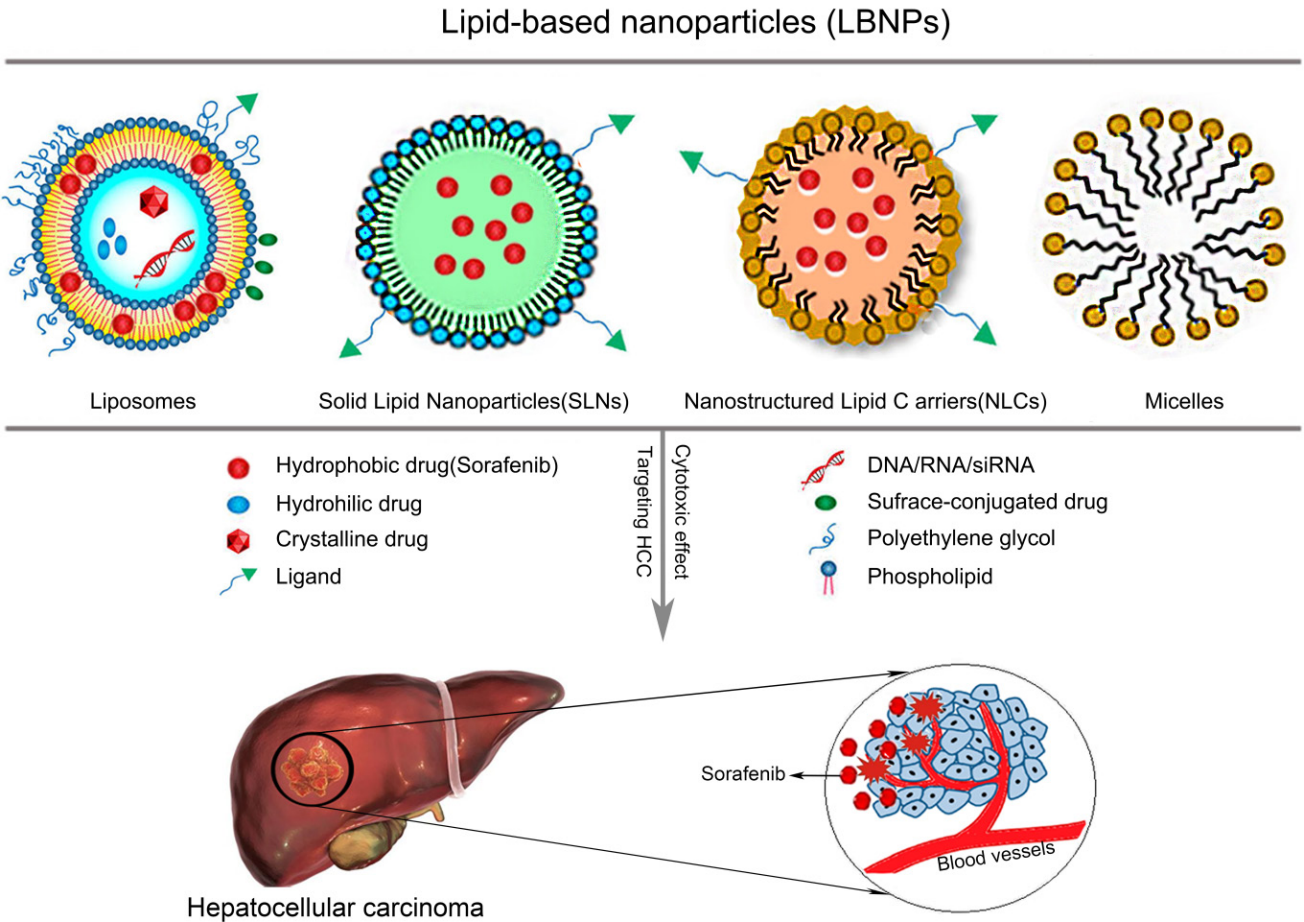
Structures of SOR-LBNPs for targeted treatment of HCC. LBNP: lipid-based nanoparticle.

Passively targeted LNPs enter tumor cells primarily through the pathways described below. First, LNPs enter tumor cells nonspecifically by integrating with cell membranes. The second pathway is receptor-mediated uptake, which is mainly observed for NLCs, and is primarily achieved using specific antibodies that recognize tumor cells 90. The third approach is to regulate drug release from NPs at specific tumor sites using external stimuli such as temperature, pH, or a magnetic field 91. The characteristics of example LNPs are described in Table 4. The structures of SOR-LNPs for targeted treatment of HCC are shown in Figure 4.

#### 3.1.2. Active targeting

Specific intermolecular recognition is the basis of active targeting. Although many active targeting strategies are available, including receptor-antibody, aptamer-ligand, and polysaccharides, active targeting of SOR-NPs is still focused on antibody- and ligand-mediated targeting 50. Notably, active targeting to tumor cells, cancer stem cells (CSCs), cancer-associated fibroblasts (CAFs), and other extravascular cells first requires extravasation, which is normally dependent on passive targeting via EPR, whereas targeting blood vessels is EPR independent. Here, we broadly divide SOR-NP active targeting strategies into targeting blood vessels, targeting cancer cells/stem cells, and targeting cancer-associated fibroblasts.

#### 3.1.3. Targeting blood vessels

Angiogenesis is a vital part of tumor development because it provides necessary oxygen and nutrients for tumor growth and a pathway for tumor metastasis. Angiogenesis inhibition can prevent or inhibit tumor growth, new tumor blood vessel formation, and metastasis. At present, VEGFR is still the main target for angiogenesis inhibition. SOR itself is able to target VEGFR, so it has a natural active targeting ability 92. For example, free SOR was combined with combretastatin A4 (CA4)-NPs to cooperatively treat HCC 57. The CA4-NPs disrupted established tumor blood vessels and induced extensive tumor necrosis; however, they also increased the expression of VEGF-A and angiogenesis. SOR reduced this VEGF-A-induced angiogenesis and further inhibited tumor proliferation. Dual vascular-targeted NPs combining VEGFR and SOR have also been reported 93. The NPs were constructed of cancer cell-macrophage hybrid membrane-coated near-infrared (NIR)-responsive hollow copper sulfide NPs encapsulating SOR and surface modified with anti-VEGFR antibody. Photothermal therapy with these NPs initially rapidly killed tumor cells, while SOR and the anti-VEGFR antibody sustained the tumor killing effect by respectively inhibiting tumor cell proliferation and angiogenesis via the Ras/Raf/MEK/ERK and PI3K/AKT pathways. Li et al. also formulated a dual-targeted delivery system by encapsulating SOR and anti-miRNA21 in arginine-glycine-aspartic acid (RGD) pentapeptide-modified reconstituted high-density lipoproteins 31. RGD and apolipoprotein A-I targeted the NPs to the tumor neovasculature and the parenchyma by binding overexpressed ανβ3-integrin and SR-B1 receptors, respectively.

Multiple mechanisms cause tumor angiogenesis, including vascular sprouting, capillary angiogenesis, endothelial cell metaplasia, and tumor vascular mimicry. Although VEGFR is the most effective strategy for targeting blood vessels, biomarkers related to angiogenesis mechanisms (e.g., extracellular matrix-related proteases, adhesion factors [integrins, selectins, cadherin], PDGFs) are significantly different, which may be the reason for the different targeting of blood vessels. In the future, it may be necessary to target tumor vascular subtypes more precisely.

#### 3.1.4. Targeting cancer cells and cancer stem cells

By targeting proteins that are highly expressed on the surface of cancer (stem) cells and less expressed on the surface of normal cells, targeted drug delivery to cancer (stem) cells can be achieved. Glypican-3 (GPC3) is a promising therapeutic target for HCC because high expression of GPC3 is associated with advanced HCC, high tumor grade, vascular invasion, short survival, and poor prognosis, and immunohistochemical GPC3 reactivity reflects tumor staging 94. Therefore, Feng et al. surface-modified LNPs with a GPC3-specific peptide 95. PLGA in the core of the LNPs provided a hydrophobic environment to encapsulate SOR. 1,2-Dioleoyl-sn-glycero-3-phosphocholine (DOPC) functioned as a lipid coating to protect the polymer matrix from water and prevent drug leakage. The NPs displayed good tumor accumulation and inhibited tumor growth. Gan et al. developed a GPC3-targeted polymer NPs (Ab-SOR-NPs) to overcome HCC resistance to SOR and the short half-life of the drug 96. The Ab-SOR-NPs were self-assembled from a biodegradable block copolymer (D-α-tocopheryl PEG 1000 succinate [TPGS]-b-PCL), Pluronic P123, and SOR and then conjugated with anti-GPC3 antibodies. The NPs showed good release of SOR in cell culture medium. Ab-SOR-NPs showed higher uptake in HepG2 cells than non-targeted SOR-NPs. MTT assays also confirmed that Ab-SOR-NPs were more cytotoxic than non-targeted SOR-NPs and free SOR, and the NPs exerted a significant inhibitory effect on tumor growth in HepG2 tumor-bearing nude mice without significant side effects. Another GPC3-targeted NPs include Ab-BEZ235-NPs, which may be beneficial to combination SOR and targeted radiosensitization of liver cells 97.

**Figure 5:**
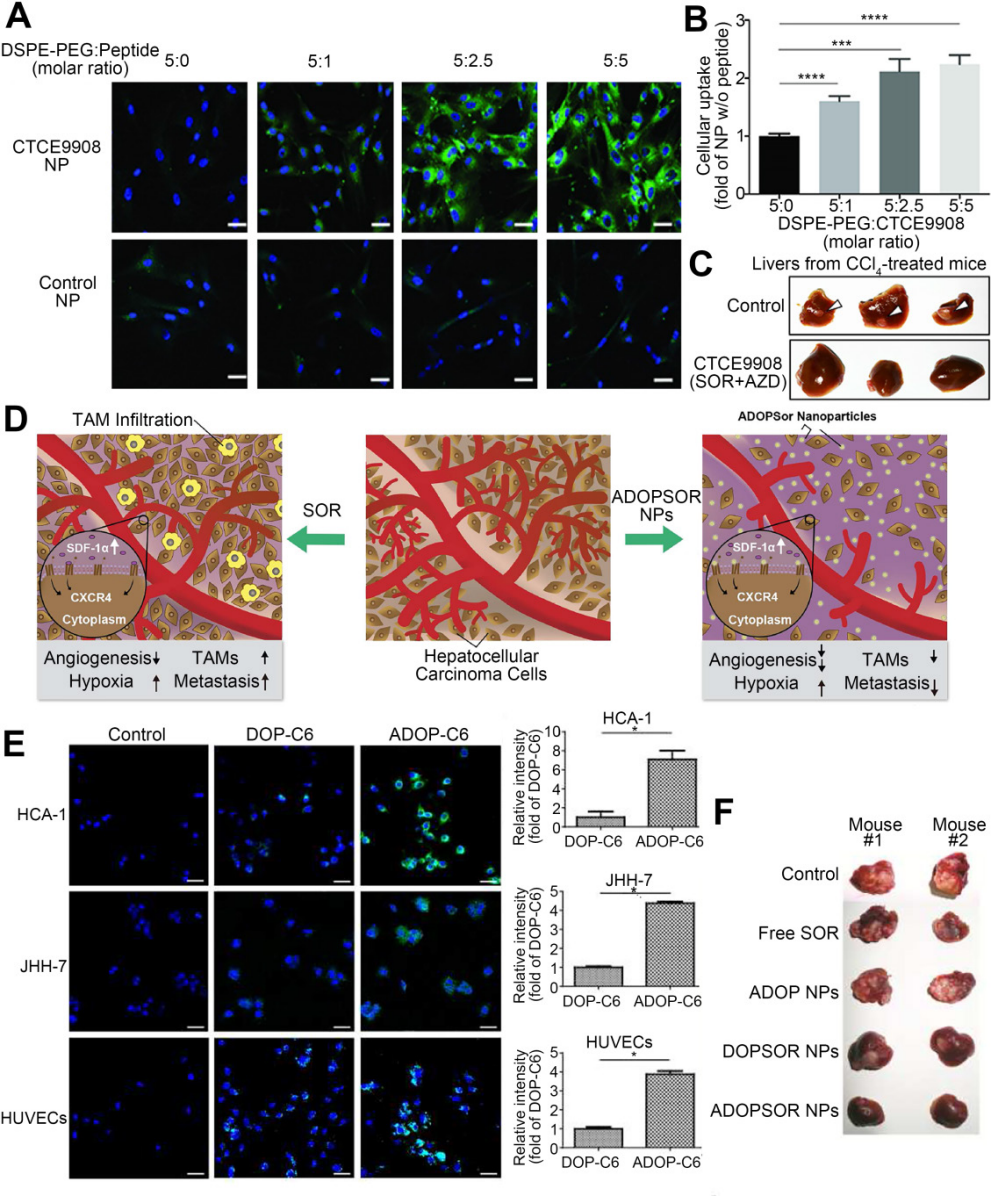
CXCR4-targeted lipid-coated PLGA NPs loaded with SOR and AZD6244 for targeted treatment of HCC. (A) Confocal microscopy images of NP uptake by activated HCC cells. (B) Quantification of (A). (C) The NPs prevented spontaneous development of HCC. (D) Schematic of the structure and mechanisms of action of the NPs. SOR increases TAMs infiltration and tumor metastasis, while NPs reduces TAMs infiltration and metastasis in the tumor microenvironment. (E) *In vitro* cellular uptake of the NPs in HCC cells and HUVECs. (F) The NPs achieved potent tumor growth inhibition. AZD6244: selumetinib; C6: coumarin 6; CTCE9908: CXCR4 antagonist; CXCR4: C-X-C chemokine receptor type 4; DOP: 1,2-dioleoyl-sn-glycero-3-phosphocholine; DSPE: distearoyl phosphatidylethanolamine; TAM: tumor-associated macrophage. Adapted with permission from 104, copyright 2018, Ivyspring International Publisher and 105, copyright 2017, Nature Publishing Group.

**Figure 6:**
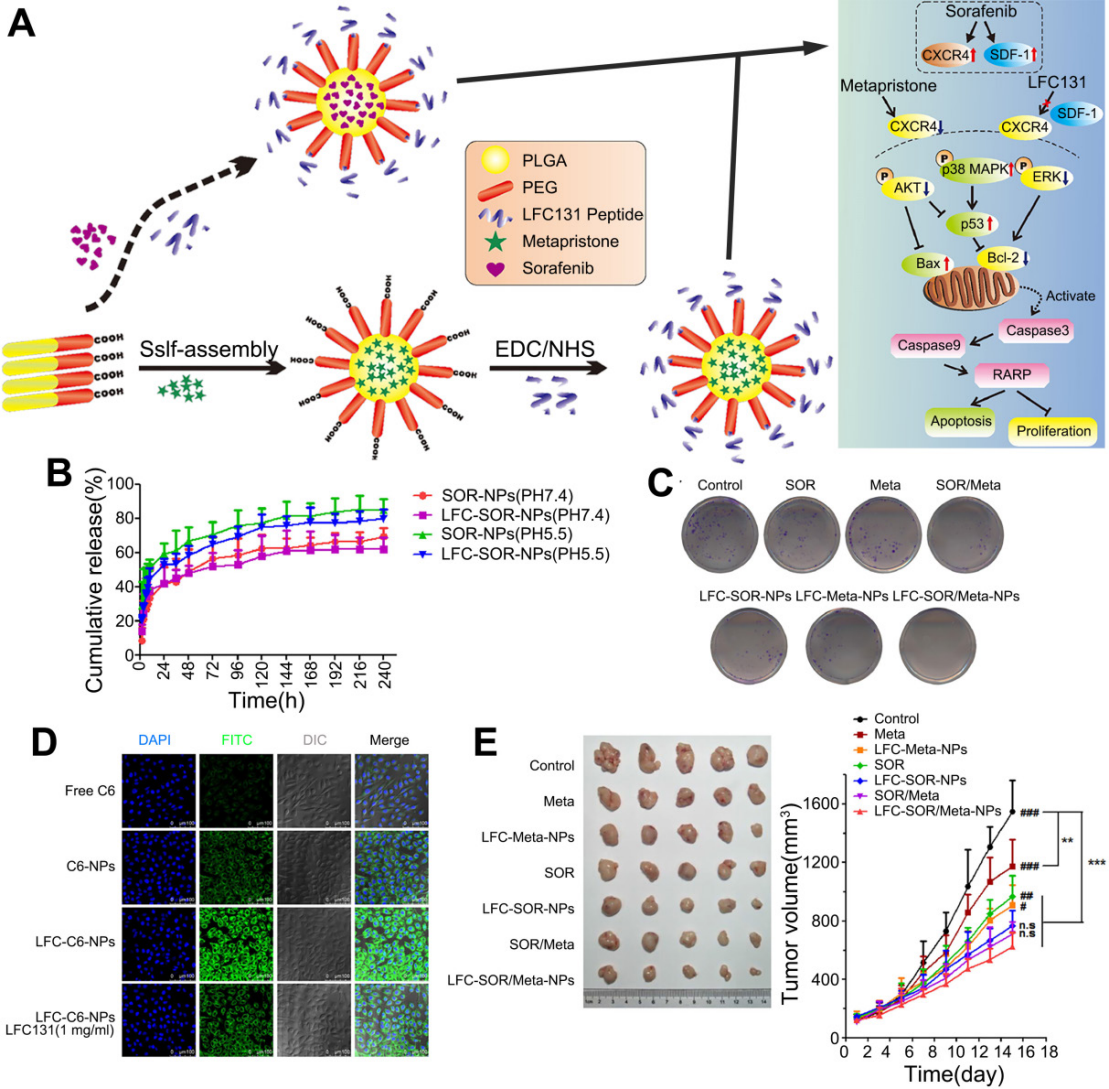
Co-delivery of SOR and metapristone in PLGA-PEG NPs for synergistic treatment of HCC. (A) Synthesis scheme and proposed mechanism of action. SOR up-regulates the expression of CXCR4 and SDF-1, while Meta and LFC131 increase tumor cell apoptosis by inhibiting CXCR4 and SDF-1. (B) Cumulative drug release profile. (C) Colony formation assay of SMMC-7721 cells. (D) Confocal microscopy images of the intracellular distribution of coumarin 6 (green) in SMMC-7721 cells after 2 h incubation with the indicated formulations. Nuclei were stained with DAPI (blue). (E) Tumor volumes and weights from the start of treatment to the endpoint. AKT: protein kinase B; Bax: BCL2 associated X, apoptosis regulator; Bcl-2: B cell leukemia/lymphoma 2; DAPI: 4',6-diamidino-2-phenylindole; LFC131: a peptide inhibitor of CXCR4; EDC: 1-(3-dimethylaminopropyl)-3-ethylcarbodiimide hydrochloride; ERK: extracellular regulated protein kinases; MAPK: mitogen-activated protein kinase; Meta: metapristone; NHS: N-hydroxysuccinimide; PARP: poly ADP-ribose polymerase; SDF-1: stromal cell-derived factor-1. Adapted with permission from 54, copyright 2019, BioMed Central.

CXCR4 is a G protein-coupled receptor that is expressed on the surface of endothelial cells, precursor cells, and pericytes and is upregulated in response to hypoxia, stress, injury, and vascular tissue injury 98. CXCR4 plays various roles in HCC progression, including promoting angiogenesis, maintaining tumor growth, inducing epithelial-mesenchymal transition, promoting invasion and spread, and helping tumor cells evade immune surveillance 99. Furthermore, abnormal overexpression of CXCR4 is closely related to poor prognosis and aggressive tumor behavior in patients with HCC 100, 101. After prolonged treatment, SOR increases hypoxia in the TME and increases the expression of CXCR4 and SDF-1α in HCC 102, 103. Thus, downregulation of CXCR4 or interventions targeting the SDF-1α/CXCR4 signaling pathway might improve SOR resistance 84. Gao et al. generated CXCR4-targeted lipid-coated PLGA NPs encapsulating the CXCR4 antagonist AMD3100 and modified with SOR to actively transport SOR to HCC cells and increase the sensitivity of HCC to SOR therapy 84. The NPs effectively delivered SOR to HCC and human umbilical vein endothelial cells (HUVECs), thereby achieving cytotoxic and antiangiogenic effects *in vivo* and *in vitro*. However, due to activation of ERK, the antitumor effect of SOR was reduced. Therefore, SOR and MEK inhibitors were co-delivered by the CXCR4-targeted NPs, which overcame the cell-independent mechanism of HCC resistance to SOR, inhibited angiogenesis, and transformed the immunosuppressive microenvironment into an immunostimulatory microenvironment. Similarly, NPs were modified with CTCE9908, a CXCR4 antagonist peptide, and loaded with SOR and the MEK inhibitor AZD6244 (Figure 5) 104, 105. By downregulating the expression of Raf/ERK and programmed cell death ligand-1 (PD-L1), the NPs promoted intracellular infiltration of cytotoxic CD8T cells, inhibited angiogenesis and the progression of liver fibrosis, and further prevented the development of fibrosis-related HCC and liver metastases, thus enhancing the antitumor effects. Prolonged use of SOR in the treatment of HCC can increase the levels of proteins involved in the SDF-1/CXCR4 axis, resulting in a poor therapeutic effect and acquired resistance. Metapristone (RU486 metabolite) is a metastatic chemopreventive agent targeting the SDF-1/CXCR4 axis 106. Zheng et al. encapsulated SOR and metapristone in a hydrophobic nucleus through hydrophobic interactions between the drugs and PLGA-PEG-COOH (Figure 6) 54. The LFC131 peptide was covalently bound to the surface of the NPs to target CXCR4, and SOR and metapristone were simultaneously delivered to CXCR4-expressing HCC cells. Given that metapristone significantly reduces the expression of CXCR4, SOR and metapristone in combination with chemotherapy synergistically inhibited the proliferation and drug resistance of HCC.

FA receptor is highly expressed in HCC, so therapeutic strategies targeting FA receptor are also common. Gao et al. grafted FA onto the surface of human serum albumin NPs encapsulating SOR 107. The NPs effectively inhibited tumor proliferation and angiogenesis without systemic toxicity. Tang et al. also prepared FA-conjugated fat-soluble chitosan/chondroitin sulfate SOR-NPs (FA-SOR-NPs) 108. Compared with untargeted SOR-NPs, the FA-SOR-NPs were significantly internalized by HCC cells due to specific interaction with the overexpressed FA receptor. The FA-SOR-NPs exerted significant cytotoxic effects at all studied concentrations with an IC50 value of 0.78 μg/mL, whereas that of SOR-NPs was 3.92 μg/mL. In addition, Zhang et al. loaded SOR and SPIONs into polymer micelles and modified the micelles with FA to target FA receptors 109. According to results from MTT assays, the average inhibition rate of HCC cells treated with the targeted NPs was significantly higher than that of the non-targeted group. The average apoptosis rates of the targeted, non-targeted, and untreated cells were 17.01%, 11.04%, and 7.89%, respectively. MRI revealed a decreased T2 signal intensity in cells treated with targeted NPs, implying that the SOR concentration in the cell medium was increased.

SOR-NPs have also been targeted to transferrin on HCC cells. For example, Malarvizhi et al. loaded DOX into a poly(vinyl alcohol) nanocore and SOR into an albumin nanoshell using continuous freezing-thawing/coagulation to form core-shell NPs targeting transferrin 110. SOR from the nanoshell inhibited aberrant oncogenic signaling involved in cell proliferation, thereby killing >75% of cancer cells.

Cancer stem cell biomarkers in HCC include CD133+, CD 49f+, CD90+, CD13, CD44, CD24, EPCAM, and SP 111, 112. To date, only CD113 and EPCAM have been used to target SOR-NPs to HCC stem cells 112, 113. Curcumin is known to inhibit the proliferation, invasion, and desiccation of CD44+/CD133+ cancer cells. In a study by Hu et al., polymer-encapsulated curcumin and SOR significantly reduced the number of CD133+ HCC cells, although the authors did not verify whether CD44+ HCC cells were also affected by this NP 112. Chen synthesized biological porous nanospheres using RNA as the building blocks and cyclodextrin as the adhesive 113. The RNA contained an aptamer of EPCAM to target delivery and siRNA for EPCAM silencing, while the cyclodextrin was loaded with insoluble SOR. The NPs effectively inhibited the activity of cells highly expressing EPCAM in *in vitro* and *in vivo* models.

#### 3.1.5. Targeting cancer-associated fibroblasts (CAFs)

CAFs are considered to be key players in cancer biology and are gradually becoming a new target for anti-HCC drugs. CAFs support the occurrence, development, and metastasis of cancer and resistance to chemical or checkpoint inhibitor treatments through various mechanisms, including angiogenesis; extracellular matrix remodeling; and the secretion of tumor-promoting and immunosuppressive cytokines, chemokines, and growth factors for active immunosuppression 114. In HCC, CAFs express α-smooth muscle actin, fibroblast activation protein, PDGFRβ, and insulin-like growth factor receptor II (IGFRII). Some studies have surface-modified non-SOR nanocarriers with PDGFRβ-binding cyclic peptides or IGFRII-binding mannose 6-phosphate, thereby effectively guiding drug delivery to activated CAFs *in vivo* 115, 116. Although the combination of natural phenolic compounds, sulforaphane, and SOR has been proven to cause CAF cycle arrest and apoptosis and inhibit the spheroidizing properties of pancreatic cancer stem cells, no SOR-NPs for CAFs have been reported. Of note, SOR targets PDGFR, so SOR-NPs may naturally regulate CAFs. However, such research has not been reported in HCC 117, 118.

### 3.2. pH-responsive SOR-NPs

pH-responsive NPs mainly use the physiological pH difference between tumor and normal tissues to deliver or release chemotherapy drugs to target tumor tissues. The pH of inflamed and tumor tissues is more acidic than that of the blood and healthy tissue. The pH is even lower in endosomes and lysosomes 119. This phenomenon prompted researchers to fabricate nanocarriers that can respond to physiological and pathological pH signals, thus triggering selective drug release in cancer cells 120. The main types of pH-sensitive SOR-NPs are liposomes, polymers, and silica NPs.

**Figure 7:**
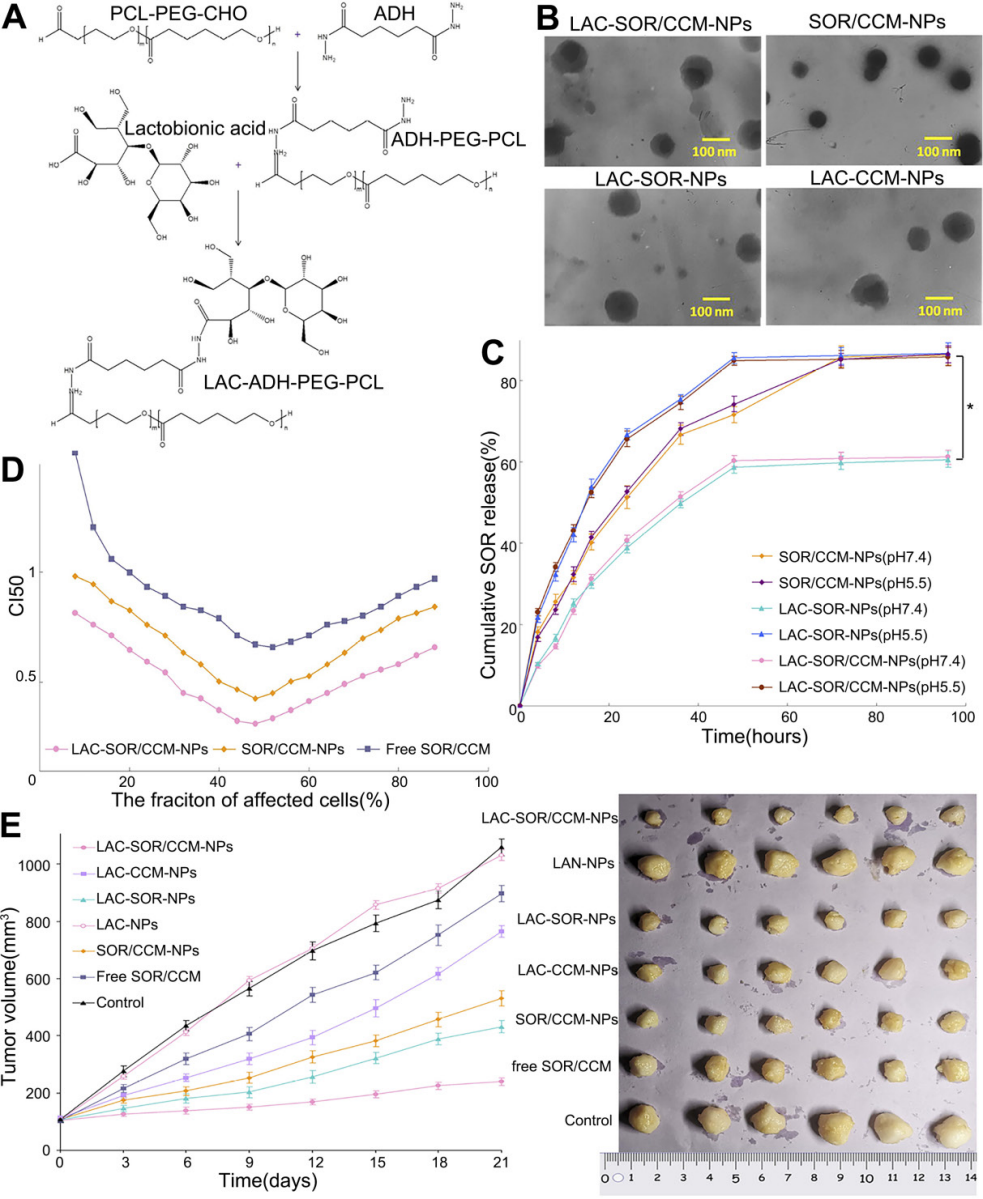
Lactosylated pH-responsive nanoparticles for co-delivery of SOR and curcumin. (A) Reaction scheme and 1H NMR spectrum of the NPs. (LAC, lactobionic acid; ADH, adipic acid dihydrazide). (B) TEM images of the NPS with and without lactosylation, revealing their different morphologies (CCM, curcumin). (C, D) Cumulative release of SOR and CCM *in vitro*. (E) Antitumor activity of the indicated NPs in a subcutaneous tumor model. ADH: adipic acid dihydrazide; CCM: curcumin; CHO: cyclohexanone oxime; LAC: lactobionic acid; NMR: nuclear magnetic resonance; PCL: polycaprolactone; TEM: transmission electron microscope. Adapted with permission from 121, copyright 2020, Dove Medical Press Ltd.

Phosphatidylethanolamine (PE) is a common component of pH-sensitive (also called acid-responsive) liposomes. When an amphiphilic molecule containing a protonatable acidic group is inserted into PE membranes, it forms a stable bilayer at physiological pH. However, at low pH, protonation of the acidic groups of the amphiphiles decreases the stability of the liposomes. Therefore, in the acidic microenvironment of tumors, unstable pH-sensitive liposomes are internalized by cells and rapidly release their cargo (Figure 7) 121. Yao et al. delivered SOR and siRNA to tumors in liposomes coated with a pH-sensitive carboxymethyl chitosan coating 122. Liposomal encapsulation effectively concentrated the siRNA and protected it from degradation by serum and RNase. Moreover, the NPs showed pH-sensitive release characteristics. *In vitro* uptake studies with fluorophore-labelled siRNA revealed a higher fluorescence intensity at pH 6.5 than at pH 7.4. *In vivo*, the NPs has a better tumor growth inhibitory effect at pH 6.5 than at pH 7.4. Zhao et al. developed pH-sensitive mesoporous silica NPs encapsulating SOR and ursolic acid 37. The NPs were modified with chitosan lactobionic acid via an acid-labile amide bond to target tumors overexpressing ASGP-R. The NPs enhanced the bioavailability of the hydrophobic drugs, effectively targeted tumor cells, and exhibited pH-responsive and sustained drug release. In addition, the NPs significantly increased apoptosis of HCC cells and decreased the levels of EGFR and VEGFR2 proteins, which are related to cell proliferation and tumor angiogenesis. In an H22 tumor mouse model, the NPs significantly reduced tumor load and volume. The greatest challenges in the future development of pH-sensitive SOR-NPs are selection, modification, and integration of relevant materials; design and safe preparation of effective pH-sensitive biomedical materials; and technical problems associated with the structural characteristics of biological materials for achieving appropriate products and clinical applications.

### 3.3. Magnet-responsive SOR-NPs

Various metal NPs have excellent magnetic properties. These additional functions provide local, accurate, and imageable application of SOR. For example, Chen et al. developed SOR-eluting PLGA microspheres for intrahepatic perfusion in rodent liver cancer models 123. The PLGA microspheres also encapsulated iron oxide NPs, enabling imaging of their intrahepatic distribution by MRI. The microspheres significantly decreased HCC cell proliferation in a dose-dependent manner. After 72 h of microsphere infusion, the tumor microvascular density significantly decreased by 35% compared with that of control tumors treated with sham surgery. These PLGA microspheres have the potential to improve the efficacy of SOR therapies. Chen et al. prepared poly(lactide-co-glycolide) microspheres co-encapsulating SOR and iron oxide NPs using a double emulsion/solvent evaporation method for local SOR administration during MRI-guided hepatic embolization in a rabbit VX2 model 124. The microspheres transferred SOR to the HCC tumor cells and reduced VEGFR levels and microvascular density within 24 h after infusion. These SOR-eluting microspheres have the potential to reduce angiogenic effects during catheter-guided embolization and improve patient tolerance to SOR.

**Figure 8:**
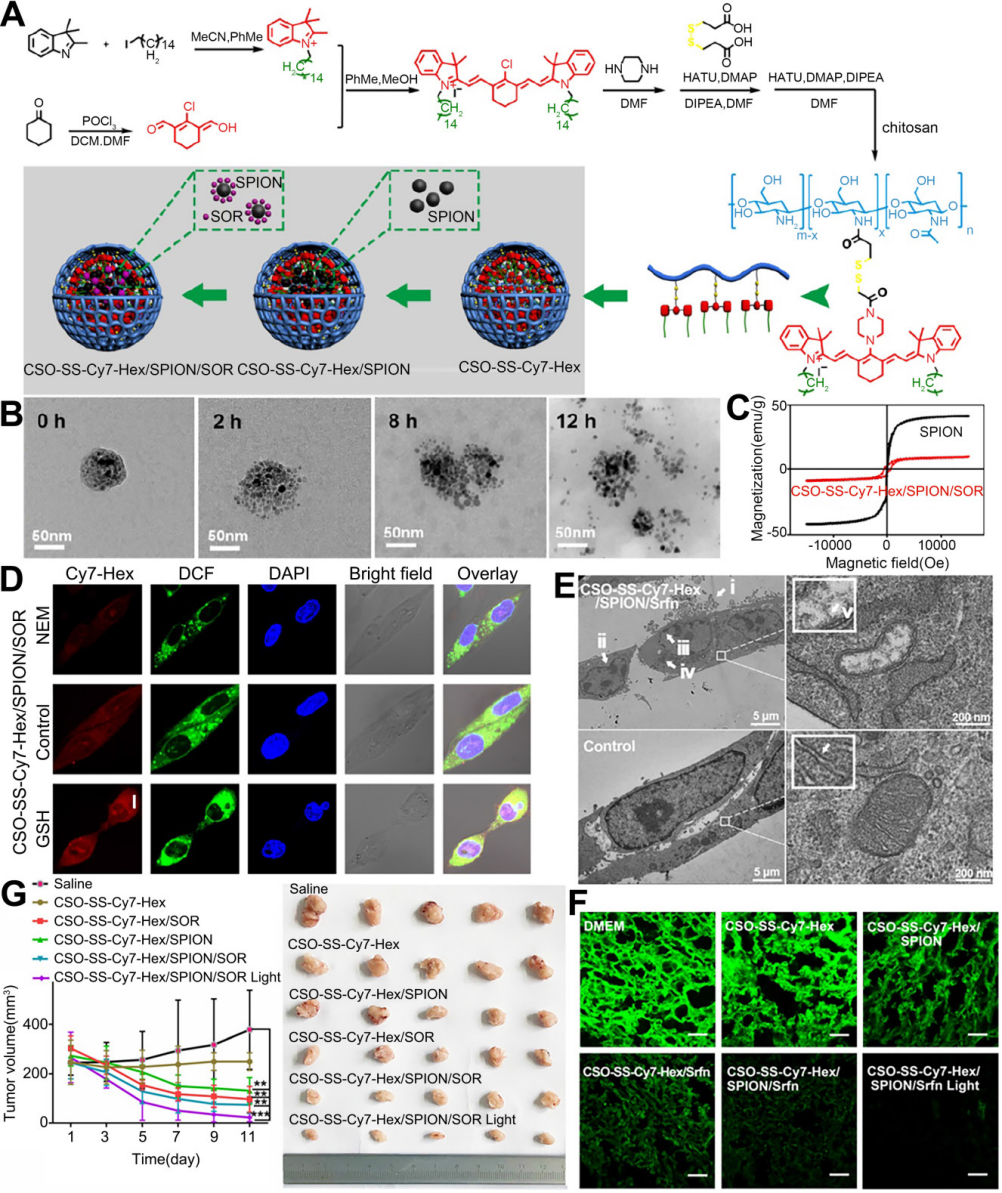
NPs encapsulating SOR, SPIONs, and Cy7-Hex for induction of lipid hydroperoxides and ferroptosis in therapy-resistant cancer. (A) Schematic illustration of the preparation of the NPs. (B) TEM images of the disassembling self-assembled NPs after incubation with 10 mM GSH for 0 h, 2 h, 8 h and 12 h. (C) Hysteresis loops of SPIONs and the NPs in self-assembly solution. (D) Confocal microscopy images of the NPs after pretreatment with 1 mM N-ethylmaleimide (NEM) and 10 mM GSH. (E) TEM image of 4T1 cells treated with the NPs for observation of ferroptosis. i-v. The mitochondria seemed smaller than normal with increased membrane density and decreased or absent mitochondrial ridges. (F) Immunofluorescence images of GPX-4 in 4T1 tumor tissues after treatment with the indicated NPs. (scale bar, 5 μm). (G) Tumor volumes from the start of treatment to the endpoint. CSO: chitosan oligosaccharide; DCM: dichloromethane; DIPEA: N,N-diisopropylethylamine; DMAP: 4-dimethylaminopyridine; DMF: dimethyl formamide; GPX-4: glutathione peroxidase 4; GSH: glutathione; HATU: O-(7-azabenzotriazol-1-yl)-N,N,N',N'-tetramethyluronium hexafluorophosphate; NEM: N-ethylmaleimide; POCl3: phosphorus oxychloride; SPION: superparamagnetic iron oxide nanoparticle; Srfn: sorafenib. Adapted with permission from 125, copyright 2019, Ivyspring International Publisher.

Another study used co-precipitation and physical encapsulation to synthesize chitosan and photosensitizer micelles encapsulating SOR and SPIONs, resulting in the formation of polyvinyl alcohol/SPIONs NPs with a size of 50-150 nm (Figure 8) 125. The NPs displayed similar or higher cytotoxicity than free SOR. Other types of magnet-responsive SOR-NPs have also been developed. For example, Xiao et al. prepared liposomes loaded with SOR and Gd 86. The solubility of SOR in the NPs was significantly increased. Additionally, the Gd provided effective MRI contrast to visualize the delivery and biological distribution of the liposomes *in vivo*. The antitumor effect of the NPs in H22 tumor-bearing mice was superior to that of SOR solution delivered orally or intravenously.

## 4. Combining SOR with other treatments for synergistic therapy

SOR can effectively inhibit the Raf/MEK/ERK signal transduction pathway to prevent tumor cell proliferation 93. Meanwhile, it also significantly inhibits VEGFR and PDGFR. However, long-term application of SOR can still lead to chemotherapy resistance. Combination of SOR with chemotherapy drugs or antitumor substances that target other signaling pathways can effectively reduce drug resistance. NPs provide an efficient and stable dosing method for SOR combination therapy 126.

### 4.1. Combination with chemotherapy

Chemotherapy drug combinations are the most common methods to overcome drug resistance in the clinic. The combination of DOX and SOR has been proven to be a feasible strategy to improve the treatment of HCC, especially in patients unsuitable for TACE 127, 128. However, there are significant differences in their pharmacokinetics and endocytosis efficiency *in vivo*, which limits their combined effects. Nanotechnology can effectively control the relative concentrations of SOR and DOX in the tissue, thus improving efficacy. For example, Zhang et al. synthesized iRGD-decorated lipid-polymer hybrid NPs with a shell-nucleus structure for the simultaneous delivery of DOX and SOR 129. The NPs showed synergistic cytotoxicity and proapoptotic activity as well as faster internalization in HepG2 cells. The NPs displayed longer circulation and greater bioavailability than free drug and significantly improved the antitumor efficacy toward transplanted liver cancer in mice. Xiong et al. designed redox-responsive NPs based on supramolecular amphiphiles formed from host-guest interactions between PEG-β-cyclodextrin and a DOX prodrug 130. The amphiphiles were self-assembled into micelles with a diameter of 166.4 nm (DOX-NPs). The DOX-NPs were successfully taken up by HepG2 cells and DOX was released into the nucleus. In addition, SOR was conveniently encapsulated into the hydrophobic core to form slightly larger SOR-DOX-NPs with a diameter of 186.2 nm. The combination NPs generated a stronger inhibitory effect on HepG2 cells *in vitro* than a physical mixture of DOX-NPs and SOR. Ye et al. modified the surface of silica NPs with low-density lipoprotein (LDL) to simultaneously deliver SOR and DOX 131. The NPs showed good stability in the physiological environment, targeting to HepG2 cells overexpressing LDL receptors, and high antitumor efficacy. In addition, Babos et al. adopted a double emulsion solvent evaporation method to co-encapsulate DOX and SOR in PLGA and PEG-PLGA NPs. The NPs exhibited good physical and chemical properties, such as a small volume, high yield, high drug entrapment efficiency, and high drug loading efficiency. The PLGA and PEG-PLGA polymers encapsulated SOR with efficiencies of 55% and 88%, respectively. SOR was sustainably released under simulated acidic tumor conditions 132.

### 4.2. Combination with natural lead compounds

The antitumor activities of natural lead compounds such as curcumin, quercetin (QT), and artemisinin have been confirmed through *in vivo* and *in vitro* experiments. Research on the combined application of SOR and these natural compounds in NPs is active. For example, Cao et al. synthesized directional self-assembled NPs using hydrophobic interactions between SOR, curcumin, and PEG derivatives of vitamin E succinic acid for synergistic treatment of HCC 133. The NPs demonstrated increased cytotoxicity and apoptosis induction in BEL-7402 and HepG2 cells compared with monotherapy and a physical mixture of SOR and curcumin. In addition, the tissue concentrations of SOR and curcumin in the gastrointestinal tract and major organs were significantly higher. The inhibitory effect of the NPs on tumor progression was also significantly stronger and the antiproliferation and antiangiogenesis effects were significantly increased. Hu et al. studied the efficacy of curcumin NPs alone or in combination with SOR 112. The curcumin NPs effectively inhibited the proliferation and invasion of liver cancer cell lines *in vitro* and blocked the growth and lung metastasis of primary tumors *in vivo*. Combination with SOR increased apoptosis and cell cycle arrest of HCC cells. At the mechanistic level, the combined action of curcumin and SOR synergistically inhibited MMP9 expression through the NF-κB/p65 signaling pathway. In addition, the combination therapy significantly reduced the number of CD133+ HCC cells, which have been described as cancer-initiating cells in HCC. Wang et al. designed RGD-modified lipid-coated NPs for the targeted treatment of HCC with SOR in combination with QT 134. As both a NP formulation and in solution, SOR combined with QT was more effective than either monotherapy. The NPs exerted the most significant inhibitory effect on tumor growth *in vivo* and cell viability *in vitro*. Wang et al. designed LDL-based targeted LNPs loaded with SOR and dihydroartemisinin 135. The NPs remarkably decreased cell viability and generated a robust antitumor response and delayed tumor growth in a xenograft tumor model.

### 4.3. Combination with siRNA

RNA interference is an effective means to inhibit disease-related gene expression and induce post-transcriptional gene silencing 136. siRNA small-molecule drugs have a simple structure, and their mechanism is highly similar to self-RNA degradation processes. Thus, it is a unique mechanism for treating tumors. Many studies have combined SOR with siRNA to known HCC therapeutic targets 113, 137, 138. For example, Shen et al. synthesized pluronic P85-PEI/TPGS nanocomplexes co-loaded with SOR and survivin shRNA (shSur) for the treatment of MDR HCC. The NPs achieved effective cellular internalization and high transfection efficiency of shSur, which significantly decreased the levels of survivin protein, arrested cell cycle, and induced apoptosis. The NPs also completely destroyed the closed capillary network formed by human microvascular endothelial cells. The NPs provided a superior antitumor effect compared with other treatments in a drug-resistant hepatoma model 139. Targeted NPs have also been developed to co-deliver SOR and siRNA. For example, LNPs loaded with SOR and midkine-specific siRNA were targeted to HCC cells using SP94 peptide 137. LNPs loaded with SOR and microRNA27a have also been targeted using GPC3 antibody 140. Zheng et al. designed and synthesized a nanodrug delivery system for SOR and siRNA against VEGF based on mesoporous silica NPs targeted to ASGP-R. The NPs delivered SOR and siVEGF to HCC simultaneously and enhanced the anticancer effects of SOR and siVEGF 138.

**Figure 9:**
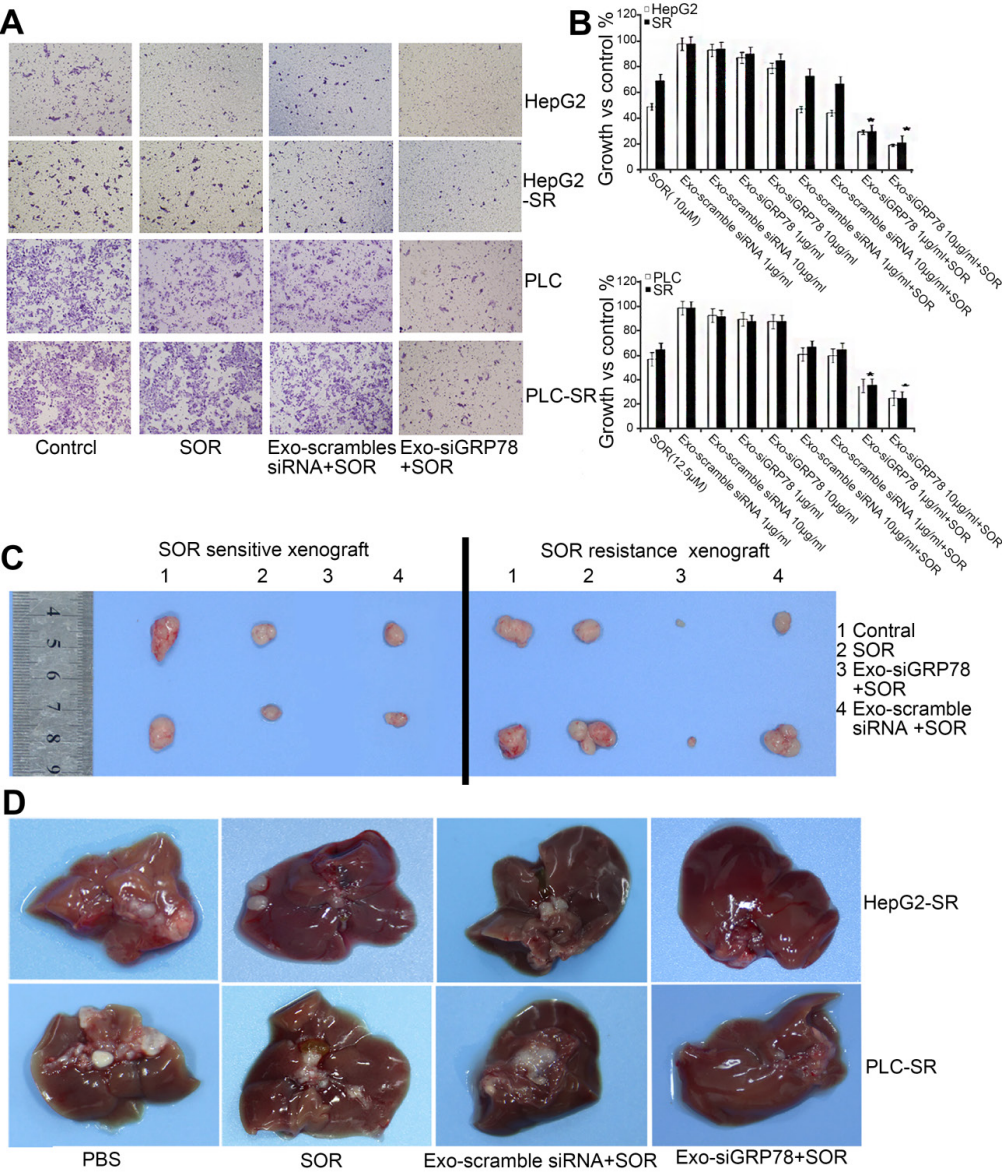
siGRP78-modified exosomes for the suppression of SOR resistance in HCC. (A) Transwell assay showing that the combination treatment inhibited the invasion ability of SOR-sensitive and -resistant cells. (B) MTT assay showing that the growth of HepG2 and PLC cells was inhibited by siGRP78-modified exosomes with or without SOR. (C) Tumor size in a subcutaneous model after the indicated treatments (control, SOR, Exo-scramble siRNA + SOR, and Exo-siGRP78 + SOR). (D) Metastasis of SOR-resistant cancer cells after the indicated treatments (control, SOR, Exo-scramble siRNA + SOR, and Exo-siGRP78 + SOR). GRP78: glucose-regulated protein, 78kDa; MTT: 3-(4,5-dimethylthiazol-2-yl)-2, 5 diphenyltetrazolium bromide; PBS: phosphate buffer saline; SR: sorafenib resistant. Adapted with permission from 151, copyright 2018, BioMed Central.

Exosomes are endocytic vesicles with a nanometer size (40-100 nm). They are initially formed in early endosomes and are subsequently secreted when multivesicular bodies fuse with the plasma membrane 141. Exosomes also contain siRNAs and microRNAs, which when delivered to target cancer cells are translated or mediate RNA silencing 142. Because exosomes can transport small molecules between cells, they are a promising therapeutic carrier for many diseases. Further, compared with exogenous nanovesicles, exosomes effectively avoid immune recognition and clearance 143. Currently, many researchers are using bone marrow mesenchymal stem cells (BM-MSCs) as a tool to obtain less immunogenic exosomes 144. GRP78 is overexpressed in many tumors and is associated with the progression of many human cancers, including lung cancer 145, colon cancer 146, gastric cancer 147, breast cancer 148, and HCC 149. Moreover, GRP78 plays an important role in HCC and promotes SOR resistance 150. Li et al. transfected BM-MSCs with siRNA against GRP78 to generate 4-120 nm exosomes containing siGRP78 (Figure 9) 151. The siGRP78-carrying exosomes were internalized by all liver cancer cells, where they targeted GRP78 and combined with SOR to inhibit growth and invasion. Moreover, the combination therapy rendered SOR-resistant cancer cells sensitive to SOR.

The mechanism of action of siRNA is similar to that of the body's own miRNA. With the further development of miRNA research, some researchers believe that directly mimicking or inhibiting miRNA may have better biological similarity and a broader antitumor targeting effect than current siRNA approaches, which provides a new direction for SOR-NPs 152. For example, a study reported the encapsulation of SOR and anti-miRNA21 NPs in RGD pentapeptide-modified recombinant HDL (RGD-rHDL/SOR/anti-miRNA21). RGD and Apo a-ion the NPs bound to αnuβ3-integrin and SR-B1 receptor overexpressed on HCC and targeted the NPs to sites of tumor angiogenesis and the parenchyma, thus achieving the accurate delivery of drugs to maximize efficacy. The simultaneous delivery of SOR and anti-miRNA21 by RGD-rHDL significantly enhanced the antitumor and antiangiogenic effects of SOR 31. calcium carbonate NPs coated with lipid and containing miR375 and SOR have been reported 153. New data obtained from some patients with HCC in clinical trials suggest that miR221 plays a role in promoting tumorigenesis in HCC by inhibiting the expression of p27 154. Cai et al. investigated the synergistic inhibition of HCC cell proliferation by the combination of SOR and anti-miR221 conjugated to gold NPs 155. The miRNA therapy increased the inhibitory effect of SOR on cell proliferation by inhibiting the miR221/p27/DNMTI signaling pathway. In addition, NPs coated with SOR and miR122 have also shown good transfection efficiency and significant inhibition of HCC cell migration and invasion 156.

### 4.4. Combination with photodynamic therapy (PDT)/photothermal therapy (PTT)

In recent decades, phototherapies 157, 158 such as PDT 159, 160 and PTT 161, 162 have attracted considerable interest for cancer therapy due to their advantages such as low damage to normal tissues, noninvasiveness, and strong therapeutic efficacy 163, 164. In PDT, irradiation of a photosensitizer (PS) produces a large number of reactive oxygen species (ROS) (e.g., singlet oxygen [^1^O_2_]) to induce tumor cell death by apoptosis 165. Thus, PDT is also a potentially effective approach to starve tumor cells of oxygen 166, 167. In PTT, irradiation of a photothermal agent converts light energy into heat to generate hyperthermia, which induces cells necrosis or apoptosis 168, 169.

NPs containing metal elements are often used in PDT/PTT. For example, Wang et al. synthesized mesoporous silica NPs with a gold nanoshell for photothermal conversion and loaded the NPs with SOR. The NPs demonstrated dose-dependent toxicity in liver cancer cells with near-infrared irradiation 170. Chen et al. constructed rough nanocapsules with a gold nanorod core and a multi-cationic mesoporous silica shell 171. The NPs were loaded with SOR and the tumor suppressor gene p53 for synergistic chemotherapy, gene therapy, and PTT. Significant cytotoxicity has also been observed in HCC cells treated with free SOR and spherical gold NPs synthesized using a citrate reduction method following laser irradiation 172. Another group designed Prussian blue metal-organic framework nanoparticles loaded with Cy5.5 and SOR and targeted with SP94 peptide. These NPs enabled multimodal imaging of SOR biodistribution and tumor targeting. SOR treatment was combined with PTT, which reduced the side effects of SOR and achieved a therapeutic effect without local tumor recurrence 173.

More commonly, PDT/PTT is achieved using small molecule PSs and photothermal agents. For example, mesoporous silica NPs loaded with indocyanine green (ICG) and SOR have been used for combination PTT and immunotherapy 174. The NPs displayed good fluorescence imaging contrast and significant photothermal tumor killing with immune enhancement in H22 tumor-bearing mice. Using π-π stacking between SOR and ICG, Wu et al. designed self-assembled NPs via a one-step nanoprecipitation method 175. The NPs were shieled with Pluronic F127 to improve their stability in aqueous solutions. The designed NPs were stable and monodisperse and demonstrated efficient photothermal generation *in vivo* and *in vitro*. The NPs rapidly entered Huh7 cells and produced large amounts of ROS under NIR irradiation, resulting in strong cytotoxicity and complete elimination of subcutaneous tumors *in vivo*. Yu et al. designed and synthesized self-assembled NPs composed of BSA, the PS zinc phthalocyanine, and SOR 176. The NPs enabled combination PDT, PTT, and chemotherapy in an HCC model. Another approach combined SOR with the PS chlorin e6 to prepare vector-free multifunctional NPs for combination antiangiogenic therapy and PDT 177. The ~152 nm nanoparticles efficiently generated ROS and heat. The NPs not only exerted substantial therapeutic effects *in vivo* but also displayed good safety and biocompatibility, indicating their broad application prospects for phototherapy and antiangiogenesis therapy with fluorescence imaging guidance. Melatonin primarily induces ROS production, leading to the mass death of FLT3-ITD AML cells. In addition, the cytotoxicity induced by SOR was significantly enhanced by redox modification, which is expected to be further studied in HCC 178.

### 4.5. Combination with immunotherapy

The liver is considered an immunotolerant tissue, a characteristic that can be attributed to the specificity of its physiological function. Therefore, hepatic sinusoidal endothelial cells express immunosuppressive molecules such as programmed cell death protein 1/ligan 1(PD-1/PD-L1) 179. High expression of PD-1 and PD-L1 has been found in patients with HCC 180, and expression of PD-L1 is associated with tumor invasiveness and poor prognosis 181. PD-L1 is a ligand that binds to PD-1 receptors on activated T cells and inactivates them, therefore HCC cells expressing PD-L1 escape the immune system and survive 182. Immunotherapy has been shown to be effective and safe for treating most solid tumors, prolonging patient overall survival (OS) and progression-free survival (PFS) and reducing the toxicity and side effects of other therapies 179. Immune checkpoint inhibitors (ICIs) are monoclonal antibodies against extracellular proteins that inhibit antitumor immune responses, such as PD-1 and CTLA-4. ICIs have been approved by the US FDA for treatment of HCC 183. Many clinical trials have attempted to evaluate the efficacy of immunotherapies in HCC, including ICIs, cancer vaccines, adoptive cell therapies, and combinations with chemoradiotherapy or targeted therapeutic NPs, with some encouraging results 179.

Immunotherapy is a promising approach for cancer treatment because it provides long-term immunity to the tumors initially treated, and NPs have been shown to improve the immune response 184. Chang et al. developed tumor-targeted MnO_2_ NPs that effectively produced oxygen and delivered SOR to HCC 185. MnO_2_ catalytically decomposed H_2_O_2_ into oxygen to alleviate hypoxia, and decomposition of Mn^2+^ ions in the TME enhanced the pH/redox response of T1-weighted MRI and SOR release. Macrophages exposed to MnO_2_ showed increased mRNA associated with immune-stimulated M1 phenotype. The NPs increased the efficacy of anti-PD-1 antibody and whole-cell cancer vaccine immunotherapy by promoting macrophage polarization and increasing the number of tumor CD8+ cytotoxic T cells. Therefore, immunotherapies based on tumor-associated macrophages (TAMs) are promising tumor treatment strategies, and drug delivery systems are advantageous for improving the co-accumulation of chemotherapy drugs and TAM re-polarization agents to tumor tissues. For example, SOR was delivered to cancer cells and IMD-0354 was delivered to TAMs using co-administered twin-like NPs 186. Compared with SOR, the nanosystem generated a synergistic antitumor effect. Recently, immunotherapeutic antibodies such as CTLA-4 and OX40 have also been used in conjunction with SOR-NPs to control tumor progression. SOR activates the Raf dimer and ERK signaling pathway, leading to downregulation of BIM and upregulation of PD-L1, thus allowing HCC to develop resistance to SOR therapy 187, 188. Therefore, compared with a single immunotherapy, multiple immunotherapies or immunotherapy combined with other treatments should be developed to improve patient OS and PFS.

## 5. Multifunctional SOR nanoplatforms

With their complicated synthesis and diverse biological functions, SOR-NPs are difficult to classify in many cases. Many SOR-NPs have good biocompatibility, high tumor killing efficiency, and a wide range of applications. For example, Duan et al. designed a pH-sensitive DOX prodrug modified with the targeting agent N-acetylgalactosamine and co-loaded it with SOR into LNPs 189. The cellular uptake efficiency and inhibitory effect of the LNPs was significantly higher than that of untargeted LNPs. The co-loaded NPs displayed a significant synergistic effect, with good tumor inhibition and low systemic toxicity. Liu et al. developed multifunctional pH-sensitive polymer SOR-NPs with surface-conjugated VEGFR antibodies and Gd-DTPA for MRI contrast 190. The NPs were spherical or elliptical in shape with a uniform size distribution (181.4 ± 3.4 nm), positive zeta potential (14.95 ± 0.60 mV), high SOR entrapment efficiency (95.02% ± 1.47%), and high SOR loading capacity (2.38% ± 0.04%). The antitumor effect of the NPs on VEGFR-overexpressing H22 tumor-bearing mice was significantly higher than that of SOR delivered orally or intravenously. By combining drug loading, imaging contrast, pH-sensitive release, and active targeting, these NPs are promising for the diagnosis and treatment of HCC. In a recent study, a multifunctional micelle was developed for simultaneous HCC-targeted delivery of SOR and tumor detection by MRI 191. The NP not only significantly improved the anticancer effects of SOR but also facilitated noninvasive tumor detection and monitoring of *in vivo* drug delivery.

Multifunctional biodegradable SOR-NPs have also been developed. For example, Tang et al. developed GPC3 antibody-targeted Ab-SOR-NPs from a copolymer of ε-caprolactone and TPGS synthesized by ring opening polymerization and Pluronic P123 192. The Ab-SOR-NPs were more readily internalized by HepG2 cells than untargeted SOR-NPs. In addition, the NPs were more stable and released more SOR in cell culture than free SOR, resulting in stronger tumor cell killing. Subsequently, the authors prepared biodegradable TPGS-b-PCL SOR-NPs using an improved nanoprecipitation method 193. The SOR-NPs had an average particle size of 122.3 nm and suitable particle size distribution, stability, drug release rate, and drug loading capacity. Compared with free SOR, the SOR-NPs were more effective at inhibiting HepG2 cell growth *in vitro* and *in vivo*. Feczko et al. loaded SOR into biocompatible and biodegradable thermally sensitive PLGA or PEG-PLGA NPs using an emulsion method and modified the surface with Gd-DTPA 194. Compared with other particle systems described in the literature, these NPs exhibited better performance. Tang et al. synthesized manganese-silica NPs for triggered release of SOR 195, 196. The manganese oxide bond in the NPs broke in the presence of glutathione (GSH), which degraded the NPs and released SOR into the TME. This reaction also induced ferroptosis of HepG2 tumor cells by consuming intracellular GSH and inhibiting GSH synthesis, thus the NPs significantly inhibited tumor growth.

## 6. Future directions and challenges

Nanomedicine has been widely explored for liver cancer therapy because it provides many approaches to treat the tumor. For various HCC chemotherapy drugs, nanomedicine has been shown to extend blood circulation, enhance targeting via peptides or aptamers, and subsequently increase cytotoxicity towards tumor cells and generally decrease tumor survival. However, despite numerous advances in cancer research, nanomedicine, and therapy, HCC remains a disease with poor prognosis. Although NPs have many advantages for treating cancer, their properties can cause problems associated with increased friction and adhesion in vessels. In addition, NPs may increase ROS production *in vivo*, leading to cytotoxicity and unpredictable reactions and interactions 175. Overcoming these limitations will provide additional opportunities to develop nanomedicines for HCC. Although preclinical research on NPs targeting HCC has increased substantially in the past few years, much more research is needed before clinical translation. Furthermore, some early phase II and phase III clinical trials of NPs showed no significant improvement in OS compared with SOR or conventional chemotherapy (Table 5) 197. Encouragingly, the latest clinical trials show that some NPs improve OS. For example, in a phase III study by Celsion, patients with HCC that were treated with a thermally sensitive liposome encapsulating DOX (ThermoDox) in combination with radiofrequency ablation (RFA) showed improved OS compared with patients treated with RFA alone (Table 5) 197.

Treatment of patients with advanced HCC and MDR remains a substantial challenge. Although MDR mechanisms in HCC are very complicated, the mainstream methods to overcome tumor MDR are to (1) downregulate the ATP-dependent drug efflux pump, (2) change the expression of programmed cell death genes, and (3) regulate the TME 198. First, increasing the intracellular drug concentration and reducing drug outflow are the primary methods to combat MDR. Overexpression of P-glycoprotein, MDR-related protein 1, and ATP-binding cassette G2 cause SOR efflux and produce MDR 199, 200. Additionally, lung resistance protein blocks the binding of anticancer drugs to nuclear targets and thus induces MDR 201. Based on the above mechanisms of tumor cell excretion, a few NPs have been developed to overcome MDR. We believe that antibody, peptide, or RNA technology can effectively block the functions of various transporters and reduce the outflow of SOR, effectively overcoming MDR and enhancing the curative effect of chemotherapy 202, 203. Second, inducing cell death in multiple ways can alleviate the resistance of tumor cells to a single killing effect. The advantage of SOR is that it not only induces apoptosis and autophagy but also induces ferroptosis, which is an iron-dependent non-apoptotic programmed death process 204. Modification of NPs with iron or ferroptosis agonists is quite simple, and a few studies have confirmed the beneficial effect of this strategy. In addition, SOR has been shown to activate CD4+ in several studies, which provides the possibility for combination with immunotherapies. This idea is not limited to combinations with PD-1/L1, as SOR could be combined with CD24/Siglec-10, a PD-1 complementary pathway 205. Finally, SOR-NPs can also be used to treat the TME. In solid tumors, tumor cells may account for only 10% of the tumor mass, and cancer stem cells are an even smaller component (1-2%). Other cells such as CAFs and TAMs are accomplices in cancer progression. SOR can also target PDGFR on CAFs, which could facilitate treatment of the TME by SOR-NPs. Treatment of the TME was demonstrated by Wang et al., who delivered SOR to tumor cells and the re-polarizer IMD-0354 to TAMs for combination chemotherapy and immunotherapy 186. These research approaches are not common for SOR-NPs and are worthy of further exploration. With further clarification of the mechanisms of MDR in liver cancer, the number of NPs developed to overcome MDR will be further increased, paving the way for transformative clinical nanomedicines. Once MDR is overcome, the prognosis of patients with HCC will significantly improve.

Nanomedicine still has the potential to become an important direction in liver cancer treatment. In order to achieve this goal, basic research should continue to explore new NPs, improve tumor targeting, and overcome MDR in tumor cells. Additionally, the therapeutic window of cancer nanomedicines should be fundamentally understood by studying nano-bio interactions. To develop clinically relevant cancer nanomedicines that benefit patients, translational research should adopt a “disease-driven” approach rather than a “formulation-driven” approach. In addition, few NPs are currently used in clinical applications, which limits overall development of nanomedicines for treating tumors. Therefore, clinical trials and applications of new NPs should be accelerated to further develop HCC nanomedicine.

## Figures and Tables

**Table 1:** Pharmacokinetic parameters of sorafenib nanoparticles *in vivo*

Investigators	Nanoparticles (NPs)	Groups	Dose	MRT (h)	Half-life (t_1/2_, h)	Time to peak (t_max_, h)	Concentration of peak (c_max_, µg/ml)	AUC_0-t_ (h·µg/ml)	AUC_0‑∞_ (h·µg/ml)	Volume of distribution (v_d_, ml/kg)	Serum clearance (ml/h/kg)
Sheng et al. 58	mPEG‑PDLLA block copolymer	Free-SOR	20 mg/kg	-	8.22(±2.35)	0.08(±0.00)	5.57(±1.65)	31.02 (±6.69)	31.64 (±6.47)	8066.11 (±4299.43)	652.09 (±147.32)
		SOR-NPs	20 mg/kg	-	12.92(±0.57)	0.08(±0.00)	453.73(±87.43)	275.39 (±26.10)	289.96 (±29.33)	1291.06 (±93.69)	69.45 (±6.99)
Khan et al. 89	PB-b-PEO	Free-SOR	15 mg/kg	61.07	42.93	4.00	3.79	143.92	11131.7	-	-
		SOR-NPs	15 mg/kg	131.43	91.38	4.00	6.37	398.69	1000217.4	-	-
Elsayed et al. 78	CNTs-SDN-MCs	Free-SOR	60 mg/kg	-	4.54(±0.74)	4.00(±0.68)	13.93(±1.98)	96.87 (±15.99)	-	930(±170)	-
		SOR-NPs	60 mg/kg	-	18.43(±2.49)	12.01 (±1.38)	24.00(±2.44)	680.60 (±69.61)	-	180(±10)	-
Tunki et al. 69	GAL-SSLN	Free-SOR	25 mg/kg	6.58 (±0.58)	4.36(±0.20)	1.17(±0.28)	6.75(±1.07)	32.59 (±2.98)	45.88 (±3.87)	-	-
		SOR-NPs	25 mg/kg	16.65 (±3.64)	16.75(±1.09)	4.00(±0.00)	28.90(±0.13)	363.09 (±32.74)	598.92 (±36.28)	-	-
Li et al. 206	ApoE-Ms-SF	Free-SOR	6 mg/kg	-	5.5	-	-	87	-	-	-
		SOR-NPs	6 mg/kg	-	6.8	8	2.8 times	160	-	-	-

(AUC = area under the curve, MRT = mean residence time)

**Table 2:** Characterization of polymer nanoparticles

Investigators	Nanoparticles (NPs)	Average particle size (nm)(±S.D.)	Zeta potential (mV)(±S.D.)	Entrapment efficiency (EE%)	Drug loading (DL%)	Drug release %	PDI
Craparo et al. 51	PHEA-EDA-PLA-GAL	101.8( ± 64.3)	+1.9 (± 2.1)	--	3.0	11.6 (24h)	--
Liu et al. 190	TPTN	181.4(±3.4)	+14.95(±0.60)	95.02(±1.47)	2.38(±0.04)	47.81(pH=7.4) 99.32(pH=5.0)	0.236
Li et al. 31	RGDrHDL/So/antimiRNA21 NPs	145.4	--	91.87	6.07	12.07(pH=7.4) 61.67(pH=5.5)	--
Shen et al. 139	SSNs	≈130	≈+27	95.16	2.03	33.5	--
Cao et al. 133	SCN	84.97(±6.03)	--	98.16(±0.23)	6.54(±0.01)	--	0.176(±0.034)
Sheng et al. 58	mPEG-PDLLA	127.3(±2.0)	-3.35(±0.42)	95(±3.2)	6.5(±0.2)	50.91(24h) 56.24(48h)	--
Monajati et al. 59	PEGylated PEI-cholesterol(F3)	106.3(pH=7.5)	+12.4(±4.3)	--	13.1(±2.65)	--	0.43 (pH=7.5)
		44.3(pH=5.5)					0.42 (pH=5.5)
Tang et al. 192	NP-SFB-Ab	115.1(±8.2)	-15.3(±0.8)	75.9	9.9	--	0.18
Xiong et al. 130	PEG-CD/AD/SF-NPs	186.2	--	--	--	76.3(pH=7.4) 92.8 (pH=5.0)	0.114
Chen et al. 123	PLG microspheres	980(±100)	--	--	1.8	65.2(72h)	--
Varshosaz et al. 207	Sorafenib loaded TMC coated EMs	127	-5.41	--	95	62(52h)	--
Babos et al. 132	PLGA RG 502H	164.6	-17.6∽-18.8	67	4.35	--	0.203
	PLGA RG 752H	142.2	--	55	5.03	88(±12)	0.123
	PEG-PLGA	177.2	--	88	5.31	48(±5)	0.076
Feczko et al. 194	Resomer®RG 752H	231.3(±30.1)	-22.2(±1.8)	76.6(±2.7)	11.2(±0.1)	90-100	0.19(±0.04)
	Resomer®RGPd5055	243.4(±40.4)	-19.5(±1.6)	75.2(±6.7)	8.9(±0.4)	50.6(±9.2)	0.15(±0.14)
Feng et al. 95	ALL*	116.8(±0.56)	-24.4(±0.20)	83.5(±3.4)	--	40-50(24h)	0.06(±0.02)
Cervello et al. 88	PBB/sorafenib	240(±7.7)	- 28.9(±5.7)	--	3.8(±0.48)	55	0.30(±0.07)
Gan et al. 96	NP-SFB-Ab	99.1(±7.3)	-16.6(±0.7)	76.3	9.5	>66.4(14d) > 75.4(30d)	0.16
Khan et al. 89	Sorafenib loaded PB-b-PEO (MW:3210 Da, SF:1mg/ml)	282.88(±22.61)	--	71.42(±11.98)	15.34(± 2.34)	20.4(24h) 36.8(48h)	0.29(±0.08)
Malarvizhi et al. 110	TfR-targeted albumin-sorafenib nano-shell	≈110	--	91	2.4	50(21d)	--
Tom et al. 208	Sorafenib loaded PVA/Fe3O4	5-15	--	76.37	--	30(8h) 66.7(80h)	--
Zheng et al. 55	Gel-SOR-LUF-SeNPs	≈100	0.493	--	--	46(3d)	--
Li et al. 206	Ms-SF	41-42	--	--	70.6-81.2	--	0.04-0.05
	ApoE-Ms-SF	37-41	--	--	70.4-86.9	--	0.03-0.10
Li et al. 209	NP-TPGS-SFB	118.3(±5.1)	3.3 (±0.4)	86.5	15.5	--	0.15
Wu et al. 210	SFB/BEZ235-NPs	114.71(±18.56)	-13(±1.45)	BEZ235:81.2 SFB:85.3	BEZ235:0.71 SFB:6.95	--	0.31(±0.08)

**Table 3:** Characterization of silica nanoparticles

Investigators	Nanoparticles (NPs)	Average particle size (nm)(±S.D.)	Zeta potential (mV)(±S.D.)	Entrapment efficiency (EE%)	Drug loading (DL%)	Drug release %	PDI
Zhao et al. 37	SO@MSN-CS-LA	210.9 (±2.8)	+7.7 (±2.6)	57.4(±2.1)	21.3(±0.9)	24.9(pH=7.4) 65.0(pH=5.5)	0.258 (±0.022)
Ye et al. 131	LDL-SLN/Sor/Dox	110.9(±6.8)	-18.3(±4.1)	--	37.1	<15(pH=7.4) 47.6(pH=5.5)	0.118
Zheng et al. 138	SO/siVEGF@MSN-LA	148.5 (±3.5)	+ 8.3(±3.5)	55.3(±2.9)	21.8(±1.6)	≈30(pH=7.4) ≈60(pH=5.5)	0.153(±0.072)
Tang et al. 195	MMSNs@SO 5(TEOS): 1(MnCl_2_)	102.6(±3.06)	-25.43	5.36(±0.64)	2.68(±0.32)	5.02±1.05(pH=7.4) 23.13±1.45(pH=5.0)	0.119(±0.01)
Yang et al. 174	(ICG+S)@mSiO_2_ ICG(1):S(3)	≈100	≈-17	--	9.75	18.8(pH=7.4) 22.36(pH=5.5)	--

**Table 4:** Characterization of lipid-based nanoparticles

Investigators	Nanoparticles (NPs)	Average particle size (nm)(±S.D.)	Zeta potential (mV)(±S.D.)	Entrapment efficiency (EE%)	Drug loading (DL%)	Drug release %	PDI
Bondi et al. 71	NLC-A NLC-B	221.0 219.0	-17.2 -18.9	100.00 58.52	18.46 10.30	79.8(24h) 50.5(24h)	0.512 0.417
Grillone et al. 67	Sor-Mag-SLNs	248 (± 113)	-23.0(±5.3)	--	--	--	0.2(±0.1)
Yao et al. 122	CMCS-SiSf-CL	200.1(±7.9)	-10.6(±1.0)	90.36(±0.63)	5.19(±0.035)	26(pH=7.4) 30(pH=6.5)	0.199(±0.31)
Xiao et al. 86	SF/Gd-liposomes	180(±1)	-7.0(±1.0)	96 (± 2)	4.3 (± 0.1)	--	0.20
Yang et al. 65	Sorafenib-LNS	164.5(±4.5)	-11.0(±0.28)	--	10.55(± 0.16)	--	0.202(±0.015)
Zhang et al. 66	HA/SF- cLNS	130.57(±14.06)	-18.1(±1.1)	--	6.8(±0.1)	33.64(72h)	0.261(± 0.004)
Benizric et al. 68	SLNs+/-	Nuclei^+^=304.4 Nuclei^-^=335.2	SLN^+^=+59.1 SLN^-^=-54.9	55-75	50	--	Nuclei^+^=0.289 Nuclei^-^=0.202
Duan et al. 189	NAcGal-DOX/SOR LNPs	121.2(±3.5)	-37.4(±3.6)	83.2(±3.3)	4.1(±0.4)	70-80(48h)	0.16(±0.03)
Liu et al. 211	LCPP NPs	102.9(±7.4)	+6.1(±3.5)	93(±6)	--	--	0.326(±0.04)
Zhao et al. 153	miR-375/Sf-LCC NPs	100.7(±12.1)	+40.37 (±3.38)	--	35.2 (±8.7)	≈40(pH=7.4) ≈72(pH=6.8)	0.116 (±0.03)
Mu et al. 212	GSI-Lip	100-150	-10-0	92.44 (±1.60)	--	≈80(48h)	0.1-0.2
Tunki et al. 69	GAL-SSLN	111.00 (±6.99)	-19.8 (±1.11)	95 (±1.8)	--	40-50(48h)	0.354 (±0.024)
Wang et al. 186	CMCS/SF-CLN CMCS/m-IMD-CLN	117.9(±3.4) 129.4(±6.8)	-21.1(±2.5) -25.5(±0.8)	-- --	5.22 (±0.25) 7.43 (±0.51)	-- --	0.277(±0.010) 0.291(±0.003)
Wang et al. 135	LD-SDN	126.5 (±1.33)	-25	94.5 (±1.62)	13.5 (±0.85)	36-38(24h) 70-80(60h)	0.135
Wang et al. 140	G-S27LN	≈165	+25	93.2 (±1.68)	≈8.95	38(pH=7.4) 76(pH=5.0)	≈0.115
Younis et al. 137	MK-siRNA/PEI core STR-R8-NPs SP94-NPs	56 (±8) 180 (±11.2) 150 (±9.4)	-8.5 (±3.55) +49 (±6.5) -10.5 (±3.75)	-- 90 (±8.25) 91.5 (±9.8)	-- -- --	-- -- --	0.270 (±0.045) 0.332 (±0.061) 0.205 (±0.035)
Zhang et al. 129	DOX+SOR/iRGD NPs	126.3(±16.4)	- 21.4(±4.6)	70.8(±2.8)	3.6(±0.05)	--	0.105(±0.016)

**Table 5:** Some clinical trial outcomes of cancer nanomedicines 197

Name/company	Formulation	Phase/Trial identifier	Condition	Primary outcome measure	Outcomes
CRLX101/Cerulean Pharma, Inc.	Nanoparticle drug-conjugates containing camptothecin (+ bevacizumab)	Phase II NCT02187302	Metastatic RCC	PFS	No statistically significant improved median PFS of treatment (3.7 mo) vs. SOC (3.9 mo)
Inotuzumab ozogamicin, CMC-544 /Pfizer, Inc.	Calicheamicin coupled to CD22-targeted antibody	Phase III NCT01564784 (INO-VATE ALL study)	ALL	CR	Statistically improved CR of treatment (80.7%) vs. chemotherapy (29.4%) in subgroup of 218 patients
				"OS"	"No statistical improved OS of treatment vs. chemotherapy"
Liposomal doxorubicin (ThermoDox) /Celsion, Inc.	Thermal-sensitive liposomal doxorubicin (+ RFA)	Phase III NCT00617981 (HEAT study)	HCC	PFS	No statistically significant improved PFS of treatment (13.97 mo) vs. RFA (13.87 mo)
				"Secondary outcome: OS"	"Statistically significant risk improvement in OS of treatment (79 mo) vs. RFA (53.6 mo) in subgroup"
NK105/Nippon Kayaku Co., Ltd. (NanoCarrier)	Polymeric micelles containing paclitaxel	Phase III NCT01644890	Metastatic /recurrent BC	PFS	No statistically significant non-inferior PFS of treatment vs. paclitaxel
CPX-351 (Vyxeos) /Jazz Pharmaceuticals (Celator Pharmaceuticals))	Liposomes containing cytarabine and daunorubicin in a 5:1 fixed ratio	Phase III NCT01696084	High-risk (secondary) AML	OS	Statistically significant improved median OS of treatment (9.56 mo) vs. SOC (5.95 mo)
OMM-398 (Onyvide)/Merrimack Pharmaceuticals, Inc.	Liposomes containing irinotecan (+ 5-FU/LEU)	Phase III NCT01494506 (NAPOLI-1 study)	Metastatic PC	OS	Statistically significant improved OS of treatment (6.1 mo) vs. 5-FU/LEU (4.2 mo)
Sacituzumab govitecan IMMU-132/Immunomedics, Inc.	SN-38 coupled to TROP2 targeted antibody	Phase II NCT01631552	Metastatic triple negative BC	Secondary outcome: ORR	Confirmed ORR of 28% in 60 patients

(5-FU: 5-fluorouracil; ALL: acute lymphoblastic leukemia; AML: acute myeloid leukemia; BC: breast cancer; CR: complete response; HCC: hepatocellular carcinoma; LEU: leucovorin; mo: months; ORR: objective response rate; OS: overall survival; PC: pancreatic cancer; PFS: progression free survival; RCC: renal cell carcinoma; RFA: radiofrequency ablation; SOC: standard of care.)

## Abbreviations

ADH: adipic acid dihydrazide
AKT: protein kinase B
ASGP-R: anti-sialic acid glycoprotein receptor
AZD6244: selumetinib
Bax: BCL2 associated X, apoptosis regulator
Bcl-2: B cell leukemia/lymphoma 2
BM-MSCs: bone marrow mesenchymal stem cells
BSA: bovine serum albumin
C6: coumarin 6
CTCE9908: CXCR4 antagonist
CA4: combretastatin A4
CAFs: cancer-associated fibroblasts
CCM: curcumin
CHO: cyclohexanone oxime
CNTs: Carbon nanotubes
CS: sensitive chitosan
CSCs: cancer stem cells
CSO: chitosan oligosaccharide
CXCR4: C-X-C chemokine receptor type 4
DAPI: 4',6-diamidino-2-phenylindole
DCM: dichloromethane
DIPEA: N,N-diisopropylethylamine
DMAP: 4-dimethylaminopyridine
DMF: dimethyl formamide
DOP: 1,2-dioleoyl-sn-glycero-3-phosphocholine
DOPC: 1,2-Dioleoyl-sn-glycero-3-phosphocholine
DOX: doxorubicin
DSPE: distearoyl phosphatidylethanolamine
EDC: 1-(3-dimethylaminopropyl)-3-ethylcarbodiimide hydrochloride
EPR: enhanced permeability and retention
ERK: extracellular regulated protein kinases
FA: folic acid
FDA: Food and Drug Administration
GO: graphene oxide
GPC3: Glypican-3
GPX-4: glutathione peroxidase 4
GRP78: glucose-regulated protein, 78kDa
GSH: glutathione
HA: hyaluronic acid
HATU: O-(7-azabenzotriazol-1-yl)-N,N,N',N'-tetramethyluronium hexafluorophosphate
HCC: hepatocellular carcinoma
HDL: high-density lipoprotein
HUVECs: human umbilical vein endothelial cells
ICG: indocyanine green
ICIs: Immune checkpoint inhibitors
IGFRII: insulin-like growth factor receptor II
LA: lactic acid
LAC: lactobionic acid
LBNP: lipid-based nanoparticle
LDL: low-density lipoprotein
LFC131: a peptide inhibitor of CXCR4
LNPs: lipid NPs
MAPK: mitogen-activated protein kinase
MDR: multidrug resistance
Meta: metapristone
MRI: magnetic resonance imaging
MSN: mesoporous silica nanoparticles
MTT: 3-(4,5-dimethylthiazol-2-yl)-2, 5 diphenyltetrazolium bromide
NEM: N-ethylmaleimide
NH2: amino
NHS: N-hydroxysuccinimide
NIR: near-infrared
NLCs: nanostructured lipid carriers
NMR: nuclear magnetic resonance
NPs: Nanoparticles
OS: overall survival
PARP: poly ADP-ribose polymerase
PBS: phosphate buffer saline
PCL: polycaprolactone
PCL: polycaprolactone
PD-1/PD-L1: programmed cell death protein 1/ ligan 1
PDGFR: platelet-derived growth factor receptor
PDT: photodynamic therapy
PE: Phosphatidylethanolamine
PEG: Polyethylene glycol
PEI: polyethyleneimine
PFS: progression-free survival
PHEA-EDA: α,β-poly(N-2-hydroxyethyl) (2-aminoethylcarbamate)-N/L-aspartamide
PLA: Polylactic acid
PLGA: poly(lactic-co-glycolic acid)
POCl3: phosphorus oxychloride
PPO: polypropylene oxide
PTT: photothermal therapy
QT: quercetin
RFA: radiofrequency ablation
RGD: arginine-glycine-aspartic acid
ROS: reactive oxygen species
SDF-1: stromal cell-derived factor-1
SLNs: solid lipid nanoparticles
SOR: Sorafenib
SPIONs: superparamagnetic iron oxide NPs
SR: sorafenib resistant
TACE: transcatheter arterial chemoembolization
TAMs: tumor-associated macrophages
TEM: transmission electron microscope
TME: tumor microenvironment
VEGFR: vascular endothelial growth factor receptor
